# Supplementary material for: LipidII interaction with specific residues of Mycobacterium tuberculosis PknB extracytoplasmic domain governs its optimal activation
Source: Nat Commun. 2019 Mar 15;10:1231. doi: 10.1038/s41467-019-09223-9 (PMC6428115; doi:10.1038/s41467-019-09223-9)
Supplement: Supplementary file 1 — Supplementary Information [file 41467_2019_9223_MOESM1_ESM.pdf]

**LipidII interaction with specific residues of *Mycobacterium tuberculosis* PknB extracytoplasmic domain governs its optimal activation**

Kaur et al.,

*Supplementary Material*

***Bacterial strains, Plasmids and reagents***

Bacterial strains used in the study are listed in Supplementary Table 8. pET28a and pENTR-D TOPO vectors were from New England Biolabs (NEB), Novagen and Invitrogen, respectively. pNit-1 vector was a kind gift from Dr. Christopher Sassetti[1] and pST-CiT vector, which was described earlier[2] was modified to replace the tet-on repressor with tet-off repressor[3]. All the reagents used for this study were of analytical grade and were obtained from Sigma Aldrich or GE Healthcare. Media components were purchased from BD Biosciences and Pristinamycin 1A was purchased from Molcan Corp. Restriction enzymes were procured from NEB and MBI Fermentas and DNA oligonucleotides were from Sigma Aldrich. All secondary antibodies were obtained from Jackson Immuno Research Laboratories.

***Supplementary Figure8. Raw Data***

***Primers used in the study***

PknB Forward Primer with NdeI site
5' CACC CAT ATG ACC ACC CCT TCC CAC CTG 3'

PknB Reverse Primer with HindIII site
5' TAGC AAGC TTA CTG GCC GAA CCT CAG CGT GAT G 3'

PknB-TM(transmembrane) with SapI site Reverse primer:
TTTTTTTT GCTCTTC A GCC GCC GAA CGT GTT GAT GGC GAT G

PASTA-2 Forward primer with SapI site
TTTTTTTT GCTCTTC A GGG CCC GAG CAA CGC GAA ATA CCC

PASTA-2 Reverse primer with SapI site
TTTTTTTT GCTCTTC A CCC AGA GCC AAC GAT GAT GATG

PASTA-1 Forward primer with SapI site
TTTTTTTT GCTCTTC A GGG GGC ATC ACC CGC GAC

PASTA-2 Reverse Primer with HindIII site
5'ATGC AAG C TTA AGA GCC AAC GAT GAT GAT 3'

PknB PASTA-4 N559D, Q560E double mutation Forward primer
GTGTCCAAGGGCGACGAATTCGTCATGCC

PknB PASTA-4 $N_{559}$→D, $Q_{560}$→E double mutation primer Reverse
GGCATGACGAATTCGTCGCCCTTGGACAC

PknB $S_{556}K_{557}$→AA (SK-AA) PASTA Domain mutation primer (in PknB WT background) Forward
CGAACTACAGGTGGCCGCGGGCAACCAATTCG

PknB $S_{556}K_{557}$→AA (SK-AA) PASTA Domain mutation primer (in PknB WT background) Reverse
CGAATTGGTTGCCCGCGGCCACCTGTAGTTCG

PknB $S_{556}K_{557}$→AA (SK-AA) PASTA Domain mutation primer (in PknB NQ-DE mutant background) Forward
CGAACTACAGGTGGCCGCGGGCGACGAATTCG

PknB $S_{556}K_{557}$→AA (SK-AA) PASTA Domain mutation primer (in PknB NQ-DE mutant background) Reverse
CGAATTCGTCGCCCGCGGCCACCTGTAGTTCG

PknB PASTA Domain Extracellular Forward Primer with BamHI site for cloning in pet28a
5'CACC GGA TCC GGC GGC ATC ACC CGC GAC 3'

Superfolder GFP Fw NdeI Primer:
5' CACC CAT ATG CGC AAG GGC GAG GAG CTG 3'

Gfp SapI reverse primer
TTTTTTTT GCTCTTC AGCT GCC GCC GTC CTT GTA CAG CTC GTC CAT GC

PknB SapI Fwd primer (GFP Fusion)
CACCTTTT GCTCTTC A AGC GGC GGC GGC AGC atg acc acc cct tcc cac ctg

PknB $T_{171}T_{173}$→AA mutation forward primer
C AAC AGC GTG GCC CAG GCA GCA GCA GTG

PknB $T_{171}T_{17}$→AA mutation reverse primer
CAC TGC TGC TGC CTG GGC CAC GCT GTT G

### *Strains used in this study*

| **Strains** | **Description** | **Source** |
|---|---|---|
| DH5α | *E. coli* strain used for cloning plasmid constructs | Invitrogen |
| BL21(DE3) codon plus | *E. coli* strain used for protein over-expression | Stratagene |
| *Rv* | H37Rv, laboratory strain of virulent *Mtb* | ATCC |
| *RvΔB* | H37Rv mutant with PknB under control of inducible pristinamycin promoter | [4] |
| *RvΔB::V* | *RvΔB* electroporated with pNit-3F vector | This study |
| *RvΔB::B* | *RvΔB* electroporated with pNit-3F-PknB construct | This study |
| *RvΔB::B-123* | *RvΔB* electroporated with pNit-3F-PknB-123 construct | This study |
| *RvΔB::B-234* | *RvΔB* electroporated with pNit-3F-PknB-234 construct | This study |
| *RvΔB::B-1212* | *RvΔB* electroporated with pNit-3F-PknB-1212 construct | This study |
| *RvΔB::B-GM* | *RvΔB* electroporated with pNit-3F-B-GM [$S_{556}K_{557}$→AA & $N_{559}Q_{560}$→DE] construct | This study |
| *RvΔB::B-M* | *RvΔB* electroporated with pNit-3F-B-M [$N_{559}Q_{560}$→DE] construct | This study |
| *RvΔB::B-G* | *RvΔB* electroporated with pNit-3F-B-G [$S_{556}K_{557}$→AA] construct | This study |
| *$mc^2$155* | Wild type *M. smegmatis* strain | ATCC, 700084 |
| *$mc^2$ΔB* | *M. smegmatis* conditional PknB knockdown strain with ATc inducible integrated PknB copy at L5 site | [5] |
| *$mc^2$ΔB::GFP-PknB* | *$mc^2$ΔB* electroporated with pNit-GFP-PknB WT construct | This study |
| *$mc^2$ΔB::GFP-PknB-GM* | *$mc^2$ΔB* electroporated with pNit-GFP-PknB GM construct | This study |
| *$mc^2$::GFP-PknB* | *$mc^2$155* electroporated with pNit-GFP-PknB WT construct | This study |
| *$mc^2$::GFP-PknB-GM* | *$mc^2$155* electroporated with pNit-GFP-PknB GM construct | This study |
| *RvΔB::V (Fig 6)* | *RvΔB* electroporated with pST-CiT vector | This study |
| *RvΔB::B (Fig 6)* | *RvΔB* electroporated with pST-CiT-PknB construct | This study |
| *RvΔB::B-TATA* | *RvΔB* electroporated with pST-CiT-PknB-TATA [$T_{171}$→A, $T_{173}$→A] construct | This study |

## *References*


1. Pandey AK*, et al.* Nitrile-inducible gene expression in mycobacteria. *Tuberculosis (Edinb)* **89**, 12-16 (2009).

2. Nagarajan SN*, et al.* Protein kinase A (PknA) of Mycobacterium tuberculosis is independently activated and is critical for growth in vitro and survival of the pathogen in the host. *J Biol Chem* **290**, 9626-9645 (2015).

3. Klotzsche M, Ehrt S, Schnappinger D. Improved tetracycline repressors for gene silencing in mycobacteria. *Nucleic Acids Res* **37**, 1778-1788 (2009).

4. Forti F, Crosta A, Ghisotti D. Pristinamycin-inducible gene regulation in mycobacteria. *J Biotechnol* **140**, 270-277 (2009).

5. Chawla Y, Upadhyay S, Khan S, Nagarajan SN, Forti F, Nandicoori VK. Protein kinase B (PknB) of Mycobacterium tuberculosis is essential for growth of the pathogen in vitro as well as for survival within the host. *J Biol Chem* **289**, 13858-13875 (2014).

| Supplementary Table 1a: Total protein intensities of PknB from the TMT quantative labelling experiment. | | | |
|---|---|---|---|
| **Sample** | **Depletion ($Log_2$)** | **3F-PknB WT ($Log_2$)** | **3F-PknB GM ($Log_2$)** |
| Replicate1 | -0.08865697 | 1.390462246 | 1.607312432 |
| Replicate2 | -0.14938223 | 1.22983952 | 1.560694042 |
| Replicate3 | -0.22059284 | 1.11959286 | 1.508375641 |
| **Sup Table 1b. Phosphorylation intensities normalized to the total PknB protein from the TMT quantative labelling experiment.** | | | |
| **Site** | **Depletion ($Log_2$)** | **3F-PknB WT ($Log_2$)** | **3F-PknB GM ($Log_2$)** |
| T171, T173 | -0.760783358 | -0.392627913 | 1.47862204 |
| T171, T173 | -0.448275238 | 0.046479816 | 0.896640623 |
| T294 | -0.811038129 | 0.317062545 | 1.678533624 |
| T294 | -1.147517067 | 0.718013623 | 1.60634268 |
| T294 | -0.996998596 | 0.727201299 | 1.658661081 |
| S305 | -1.467736383 | 0.980817808 | 3.01397756 |
| S305 | -1.521781303 | 0.868445276 | 2.185081107 |
| S305 | -1.550434591 | 0.978691936 | 2.237399509 |
| T309 | -1.280937563 | 1.495892798 | 2.938841203 |
| T309 | -1.361074831 | 2.476359467 | 3.479190078 |
| T309 | -1.094139754 | 1.97633156 | 2.89455114 |

**Supplementary Table 2:** Mean normalized $Log_2$ phosphopeptide intenstity ratios. These values were used for generation of heatmap (Figure 7a).

| S.No | Phosphopeptides (Phosphopeptides) | Gene Number | Gene Name | Depletion ($Log_2$) | 3F-WT ($Log_2$) | 3F-GM ($Log_2$) |
|---|---|---|---|---|---|---|
| 1 | LGIPQIsTGELFR(S30) | Rv0733 | adk | -1.128123353 | -2.306694226 | -0.321561442 |
| 2 | APAEtQAQR(T150) | Rv0251c | hsp | -1.028545672 | -2.073393844 | -1.88049134 |
| 3 | FEQSSNLHtGQFR(T116) | Rv0020c | fhaA | -0.389332853 | -1.83625844 | -0.810650595 |
| 4 | IDTsGLPAVGDDATVPR(S73) | Rv2921c | ftsY | -1.362618269 | -1.799975989 | -0.431915769 |
| 5 | LGGGAPTVEEsR(S230) | Rv3814c | Rv3814c | -0.071701108 | -1.614550149 | -0.299208357 |
| 6 | DQTSDEVTVEtTSVFR(T21) | Rv1827 | garA | -0.378167452 | -1.301596482 | -0.547386246 |
| 7 | DVLAVVsK(S99) | Rv3418c | groES | -0.370682545 | -1.189414585 | 0.514423135 |
| 8 | ADDSPtGEMQVAQPEAQTAAVATVER(T173) | Rv2536 | Rv2536 | -0.668678032 | -1.109133129 | -0.158624606 |
| 9 | sGIIPISR(S88) | Rv2778c | Rv2778c | -2.162747997 | -1.060971513 | 0.286481241 |
| 10 | VADNtGAK(T14) | Rv0714 | rplN | -0.440077072 | -0.913343142 | 1.693559489 |
| 11 | sFVAAPGR(S20) | Rv0341 | iniB | -0.424649803 | -0.893177676 | 0.286564406 |
| 12 | ITQIYEGtNQIQR(T375) | Rv3274c | fadE25 | -2.362982285 | -0.773005715 | -0.157448466 |
| 13 | TTESDTPtEVIR(T209) | Rv2536 | Rv2536 | -0.265463705 | -0.580773746 | 0.228276131 |
| 14 | GGYPPETGGYPPQPGyPRPR(Y215) | Rv0020c | fhaA | -0.189405841 | -0.494167986 | 1.175654461 |
| 15 | VVEEARtDAER(T78) | Rv1307 | atpH | -1.515582024 | -0.480462401 | 0.846122611 |
| 16 | tGGLVMVR(T202) | Rv0701 | rplC | 0.296896832 | -0.457335597 | 1.629712142 |
| 17 | SVLAtLDER(T261) | Rv2710 | sigB | -1.53157515 | -0.444910435 | 1.447557034 |
| 18 | AVVsGAQR(S90) | Rv2986c | hupB | 0.158295771 | -0.423657923 | 2.775386017 |
| 19 | EAPtEVIR(T197) | Rv2536 | Rv2536 | -0.462888892 | -0.412088696 | 0.515968467 |
| 20 | AADtDVFSAVR(T160) | Rv2536 | Rv2536 | -0.840369802 | -0.387371311 | 0.538501776 |
| 21 | MPsGEIR(S179) | Rv0704 | rplB | -0.614500148 | -0.32410968 | 1.92222591 |
| 22 | LPQAtGASTQPQLSR(T75) | Rv0384c | clpB | -0.546795556 | -0.318473452 | 1.397666166 |
| 23 | SQVGAtITHR(T205) | Rv0046c | ino1 | -1.49416646 | -0.31416488 | 0.083603757 |
| 24 | DDQPLADtANQVALAIR(T579) | Rv1133c | metE | -1.41879986 | -0.280737677 | 0.536633043 |
| 25 | VDEEtGK(T91) | Rv0715 | rplX | 0.299817451 | -0.244530984 | 2.199778803 |
| 26 | TIAyDEEAR(Y7) | Rv0440 | groEL2 | -0.818259242 | -0.238375084 | 1.563176256 |
| 27 | YsTDLTAR(S165) | Rv0384c | clpB | -1.124317891 | -0.223865605 | 2.411205142 |
| 28 | ItTtEPK(T35; T37) | Rv3456c | rplQ | 0.1834629 | -0.115971098 | 2.06081066 |
| 29 | tVDALMR(T83) | Rv0700 | rpsJ | -0.158788873 | -0.105570924 | 2.79338692 |
| 30 | TtLtAAITK(T27; T29) | Rv0685 | tuf | -2.171301376 | -0.069330572 | 1.348069923 |
| 31 | ADDSPtGEMQVAQPEAQTAAVAtVER(T173; T190) | Rv2536 | Rv2536 | -0.450484939 | -0.040652794 | 0.882931543 |
| 32 | AALGGsDISAIK(S558) | Rv0350 | dnaK | -0.615291889 | -0.008229699 | 1.852434688 |
| 33 | ESyGVGVER(Y65) | Rv2904c | rplS | 0.077033423 | 0.010460937 | 2.507469029 |
| 34 | DNtDQFNTR(T940) | Rv0284 | eccC3 | -1.634421791 | 0.010669857 | 1.344571524 |
| 35 | IQEGsGLSK(S479) | Rv0350 | dnaK | -0.731240935 | 0.040270087 | 1.852596792 |
| 36 | EIHADLLEHtEGE(T107) | Rv1390 | rpoZ | -0.470423232 | 0.101012799 | 2.107561923 |
| 37 | LGAVSYEtDR(T18) | Rv2050 | Rv2050 | 0.087969287 | 0.144908211 | 2.050186025 |
| 38 | VAVtGEVLGHTK(T62) | Rv2442c | rplU | 0.033142166 | 0.192658741 | 3.13408628 |
| 39 | sAALLTR(S209) | Rv2890c | rpsB | -1.087971718 | 0.1991221 | 2.244941311 |
| 40 | AAtEELEGYR(T131) | Rv1129c | Rv1129c | -0.616156852 | 0.200218242 | 2.249259842 |

| | | | | | | |
|---|---|---|---|---|---|---|
| 41 | AHDEDsVAGIGDR(S104) | Rv0710 | rpsQ | 0.450933666 | 0.308736191 | 2.591857778 |
| 42 | SQQsFEEVSAR(S114) | Rv0475 | hbhA | -0.173314077 | 0.337768352 | 3.128440531 |
| 43 | EHQsDIEV(S484) | Rv0647c | Rv0647c | -0.303321542 | 0.347617012 | 1.735621103 |
| 44 | GLAALIPTGPADGESGPPtLGPR(T32) | Rv3917c | parB | -1.290592804 | 0.398236621 | 1.697308972 |
| 45 | EEPsLIEINSER(S47) | Rv2909c | rpsP | -0.008535164 | 0.415891587 | 3.343972295 |
| 46 | VELQDGtVTGDYDAR(T96) | Rv1558 | Rv1558 | -1.520185927 | 0.453909603 | 2.982699753 |
| 47 | VtMSDVR(T184) | Rv0469 | umaA | -1.912472421 | 0.4599507 | 1.708632432 |
| 48 | AtDLTVAK(T56) | Rv3914 | trxC | -0.275337977 | 0.465765961 | 3.583597495 |
| 49 | QVLDtLVDAGVAR(T175) | Rv1265 | Rv1265 | -0.806607264 | 0.501138528 | 1.801031635 |
| 50 | EQsAAIR(S80) | Rv3597c | lsr2 | -0.70124662 | 0.51136775 | 3.530065772 |
| 51 | LASGEPAsGPASNSPAR(S196) | Rv0042c | Rv0042c | -0.153958744 | 0.515599568 | 2.859214688 |
| 52 | DYLDEtGGR(T256) | Rv3410c | guaB3 | -1.375797379 | 0.516091039 | 2.097789116 |
| 53 | VINANLtGAFR(T114) | Rv1483 | fabG1 | 0.365716845 | 0.517648728 | 2.568065624 |
| 54 | VyNTAEQNAANAGK(Y284) | Rv2187 | fadD15 | -1.177844293 | 0.524482477 | 1.789250326 |
| 55 | StVEGANDALAR(T90) | Rv3852 | hns | -0.65218215 | 0.562415653 | 2.158641738 |
| 56 | GGPDPQGGsDPR(S196) | Rv0020c | fhaA | 0.173189655 | 0.571572675 | 2.142939822 |
| 57 | EITALEGEVATLsER(S146) | Rv1626 | Rv1626 | 0.129994517 | 0.581474618 | 3.058565323 |
| 58 | DIYGAALGYQVDAtNLQR(T182) | Rv1593c | Rv1593c | -0.854152037 | 0.587032883 | 2.498316888 |
| 59 | YANAIGsAELAESSVQGR(S203) | Rv2744c | 35kd_ag | -0.842184458 | 0.638168294 | 2.185082465 |
| 60 | TLVSNLVTGVTQGYtTK(T85) | Rv0719 | rplF | -0.111607257 | 0.643571939 | 3.029693357 |
| 61 | DTTyLYR(Y72) | Rv2534c | efp | -0.175544234 | 0.643914509 | 2.717045928 |
| 62 | VTSELSHtQGELR(T68) | Rv3105c | prfB | -0.682094264 | 0.649219975 | 2.094723318 |
| 63 | DAIITDtAITER(T103) | Rv0474 | Rv0474 | -1.369509995 | 0.690497736 | 2.67762055 |
| 64 | MNELVDtTEMYLR(T7) | Rv2711 | ideR | -0.763090981 | 0.701756844 | 2.506934104 |
| 65 | TyLESQLEELGQR(Y226) | Rv2145c | wag31 | -0.834645243 | 0.70968735 | 2.761180938 |
| 66 | sGVSNPYLSQVER(S44) | Rv0474 | Rv0474 | -1.113210871 | 0.71474526 | 2.929959673 |
| 67 | LPGLESPEEEsAAR(S107) | Rv1265 | Rv1265 | -0.737218861 | 0.72153176 | 2.334912385 |
| 68 | VEKDPENPtVVLtVR(T213; T217) | Rv3246c | mtrA | -1.043305829 | 0.736455654 | 2.524923653 |
| 69 | GDSVTItGFGVFEQR(T45) | Rv2986c | hupB | -0.051639278 | 0.787725943 | 3.334438593 |
| 70 | AQyLSGR(Y612) | Rv1925 | fadD31 | -2.163423967 | 0.788184829 | 1.48406619 |
| 71 | LDsGEQLK(S66) | Rv1334 | mec | 0.825213568 | 0.79758924 | 3.667786774 |
| 72 | TISEAGQAMAStEGNVTGMFA(T86) | Rv3875 | esxA | -0.383727809 | 0.804502211 | 2.604874943 |
| 73 | AFEAGEPQAsGK(S88) | Rv0566c | Rv0566c | 0.597078699 | 0.828254524 | 3.977904924 |
| 74 | TVTPEGDtK(T116) | Rv1443c | Rv1443c | 0.337100421 | 0.837962695 | 2.786961322 |
| 75 | AAVtAVSDAVR(T580) | Rv0384c | clpB | -0.935954923 | 0.873541955 | 3.00967902 |
| 76 | tALLNAAGEVELAK(T37) | Rv2710 | sigB | -1.635365134 | 0.903644762 | 2.615894598 |
| 77 | SPDEFsGK(S170) | Rv0815c | cysA2 | -0.755019344 | 0.910128763 | 2.720822493 |
| 78 | TGASVVGPVPLPtEK(T44) | Rv0700 | rpsJ | 0.374622762 | 0.910333464 | 3.832709669 |
| 79 | AAEGyLEAATSR(Y87) | Rv0475 | hbhA | 0.002058964 | 0.913219633 | 3.669092829 |
| 80 | LLEGEtAK(T558) | Rv0384c | clpB | -0.690513245 | 0.939834059 | 3.682365729 |
| 81 | VPSAEEMADsLR(S713) | Rv1133c | metE | -1.160094411 | 0.94086685 | 2.694350721 |
| 82 | VDSGVEtGSVIGGQFDSMLAK(T383) | Rv3285 | accA3 | -1.115317082 | 0.943135938 | 2.857000644 |
| 83 | LSGyLAAAK(Y337) | Rv0147 | Rv0147 | -2.180572863 | 0.951662847 | 2.278460277 |
| 84 | tLDEIGQVYGVTR(T488) | Rv2703 | sigA | 0.116097582 | 0.964660633 | 3.202402296 |
| 85 | tIVVELEDR(T73) | Rv0710 | rpsQ | -0.229344475 | 0.96604892 | 3.380301837 |
| 86 | AAGVDVDPLDtGAPVAIASAAAVSGLR(T133) | Rv2096c | pafB | -0.354035361 | 0.971787485 | 2.959119428 |
| 87 | tTPSIVAFAR(T35) | Rv0350 | dnaK | -0.44692518 | 0.972225307 | 3.939215528 |
| 88 | RIDPEtGEVR(T28) | Rv0351 | grpE | -0.436219743 | 0.996781803 | 2.595606536 |
| 89 | AAVPQPsEK(S69) | Rv0250c | Rv0250c | -1.213708729 | 1.007787147 | 2.324867142 |
| 90 | SLNtVPFQIAR(T147) | Rv3458c | rpsD | 0.704808635 | 1.018622448 | 3.698255001 |
| 91 | ILDsGGR(S54) | Rv1298 | rpmE | -0.346072302 | 1.028754061 | 4.198785482 |
| 92 | DLtEEQLIHLR(T49) | Rv3460c | rpsM | -0.323282689 | 1.029370213 | 3.778102199 |
| 93 | tGIAQVATVSSR(T141) | Rv3417c | groEL1 | -0.859984901 | 1.029673006 | 2.81684682 |
| 94 | DyIEANLK(Y59) | Rv3460c | rpsM | -0.114346733 | 1.071202259 | 4.094828363 |

| | | | | | | |
|---|---|---|---|---|---|---|
| 95 | GSLDtQGVK(T104) | Rv0682 | rpsL | -0.497073958 | 1.072662397 | 3.24750367 |
| 96 | tLDQIGK(T283) | Rv2710 | sigB | -1.36358639 | 1.077323404 | 3.152646176 |
| 97 | GVLDEAtGR(T153) | Rv1488 | Rv1488 | -1.279929335 | 1.100184529 | 2.495272355 |
| 98 | GVGyVLR(Y241) | Rv0757 | phoP | -0.944935927 | 1.102275188 | 3.315588599 |
| 99 | QDAtVEVAIR(T41) | Rv0641 | rplA | -0.65102661 | 1.135069139 | 3.231507882 |
| 100 | VDAtRVEQR(T181) | Rv3457c | rpoA | -0.86103971 | 1.139325461 | 2.872109466 |
| 101 | LtSIALPR(T111) | Rv0716 | rplE | 0.140895974 | 1.139914076 | 4.286390263 |
| 102 | NQAVtNVDR(T60) | Rv0350 | dnaK | -0.975436072 | 1.140299946 | 3.073934409 |
| 103 | DQPFItGER(T250) | Rv0467 | icl1 | -2.022854105 | 1.140573584 | 1.756721344 |
| 104 | AAAyLQAQGQAK(Y247) | Rv1488 | Rv1488 | -1.527184053 | 1.175546823 | 2.801227999 |
| 105 | MDLtDDSWAAVR(T116) | Rv0639 | nusG | -0.778437341 | 1.193308075 | 3.369012285 |
| 106 | IAGsQVLK(S594) | Rv0684 | fusA1 | -1.274963436 | 1.194673513 | 2.556237073 |
| 107 | LAADVADVPtVVIAR(T223) | Rv0467 | icl1 | -1.78998422 | 1.206168039 | 2.332763078 |
| 108 | AGILEPSETsQVR(S93) | Rv0474 | Rv0474 | -1.419921407 | 1.23382812 | 3.15770163 |
| 109 | DAIITDtAItER(T103; T106) | Rv0474 | Rv0474 | -1.342737486 | 1.2602227 | 3.404464861 |
| 110 | AEASIETPtPVQSQR(T60) | Rv2094c | tatA | -0.009495478 | 1.291573062 | 3.010476115 |
| 111 | SETFTTADDNQPsVQIQVYQGER(S409) | Rv0350 | dnaK | -0.640084525 | 1.298182346 | 3.518799337 |
| 112 | SNEILAAtGIDR(T37) | Rv3460c | rpsM | 0.05516429 | 1.318616274 | 4.218089789 |
| 113 | VtETLLK(T127) | Rv0440 | groEL2 | -0.705459387 | 1.32874573 | 3.195851889 |
| 114 | FTSEAALFGNDLAtQPNYPAEIR(T61) | Rv2455c | Rv2455c | -0.785455517 | 1.331770556 | 3.262073385 |
| 115 | DETTIVEGAGDtDAIAGR(T337) | Rv0440 | groEL2 | -0.399621187 | 1.345267326 | 3.039975608 |
| 116 | AQEIMTELEIAPtR(T169) | Rv1388 | mihF | 0.445640285 | 1.346529452 | 3.407245074 |
| 117 | AEGYVDQAVELtQEALGTVASQTR(T133) | Rv0475 | hbhA | 0.039765864 | 1.355651752 | 3.992177381 |
| 118 | TAMsAVDDLR(S212) | Rv1265 | Rv1265 | -0.894044774 | 1.372148977 | 2.462711336 |
| 119 | tVVAANDTK(T48) | Rv1642 | rpmI | 0.617729486 | 1.394189612 | 4.207725236 |
| 120 | AMILTAEGtR(T198) | Rv1488 | Rv1488 | -1.401960306 | 1.413327479 | 2.778004428 |
| 121 | YPHQPAPtGA(T264) | Rv1463 | Rv1463 | -0.39701753 | 1.451884901 | 3.476299366 |
| 122 | TLLSSIPGAAVtSIR(T52) | Rv3457c | rpoA | -1.122206701 | 1.45526077 | 3.496764417 |
| 123 | tLEENQK(T41) | Rv3648c | cspA | 0.29580753 | 1.460719928 | 3.779828931 |
| 124 | VPGyAPQGGGYAEPAGR(Y388) | Rv0020c | fhaA | -0.400784793 | 1.477677464 | 3.678650319 |
| 125 | QEIDQtRDQLAATIDSLAER(T16) | Rv2520c | Rv2520c | -1.753158453 | 1.491505891 | 2.977023547 |
| 126 | QYAEyVK(Y31) | Rv0707 | rpsC | -1.686252284 | 1.503469726 | 2.736413967 |
| 127 | AEAsIETPtPVQSQR(S55; T60) | Rv2094c | tatA | -0.370095698 | 1.509749048 | 3.276080188 |
| 128 | VVVELSPyDLSR(Y61) | Rv3462c | infA | -0.245993181 | 1.519471275 | 3.947650974 |
| 129 | ETPVELtFGQVSK(T231) | Rv0639 | nusG | -0.662080756 | 1.524168167 | 4.567149698 |
| 130 | FGLtDGQPR(T482) | Rv2703 | sigA | -0.82717448 | 1.538848672 | 3.859620619 |
| 131 | VITQTGtIAQSGR(T199) | Rv1593c | Rv1593c | -0.315459809 | 1.573992252 | 3.727150553 |
| 132 | sMSELAAPGNTPSLDEVR(S173) | Rv2744c | 35kd_ag | -0.166945223 | 1.574439598 | 3.143268972 |
| 133 | FtDSQLR(T217) | Rv1593c | Rv1593c | -1.162025401 | 1.575317226 | 3.82503211 |
| 134 | AGQPVSLSPTEFtLLR(T180) | Rv0757 | phoP | -0.094265121 | 1.577069418 | 3.65875517 |
| 135 | NDPtQQIPK(T234) | Rv1886c | fbpB | 0.726974464 | 1.581317571 | 4.322262372 |
| 136 | DDtAVLVTLTAR(T226) | Rv3200c | Rv3200c | -0.811683875 | 1.585498873 | 2.915973906 |
| 137 | QTGGsGQFAK(S509) | Rv0684 | fusA1 | -0.596567541 | 1.602668097 | 3.279774122 |
| 138 | DAEsDEVLGK(S133) | Rv1388 | mihF | 0.175267638 | 1.615773643 | 4.770047626 |
| 139 | AQEIMTELEIAPtR(T169) | Rv1388 | mihF | 0.707037399 | 1.630395267 | 3.806896289 |
| 140 | YTAPEIsAR(S92) | Rv0350 | dnaK | -0.565287217 | 1.632318523 | 4.368230612 |
| 141 | DQLAAtIDSLAER(T23) | Rv2520c | Rv2520c | -1.410779085 | 1.636891802 | 3.102693316 |
| 142 | EVALLPFTsSVR(S81) | Rv0055 | rpsR1 | 0.522193506 | 1.637078846 | 4.379455835 |
| 143 | NEGyISDFR(Y45) | Rv0718 | rpsH | -0.269410163 | 1.648133991 | 4.602312264 |
| 144 | TDAAtLAQEAGNFER(T10) | Rv3874 | esxB | -0.558434225 | 1.651325265 | 3.373335182 |
| 145 | LEAEsK(S114) | Rv2244 | acpM | -0.251587057 | 1.659790017 | 4.074228237 |
| 146 | YGGtEIK(T77) | Rv3418c | groES | -1.041668572 | 1.661610299 | 3.554351376 |
| 147 | tAIVEGLAQR(T213) | Rv0384c | clpB | -0.714246302 | 1.680239022 | 4.071438864 |
| 148 | VTVtAADLR(T48) | Rv0300 | vapB2 | -0.529912684 | 1.68540779 | 3.793759183 |

| | | | | | | |
|---|---|---|---|---|---|---|
| 149 | ADLLVVGNVGLStIAGR(T119) | Rv1636 | TB15.3 | -0.220129161 | 1.690029935 | 3.708260744 |
| 150 | tGETVK(T65) | Rv2986c | hupB | 0.7287785 | 1.691743353 | 4.763452062 |
| 151 | ELEAMEQtGGFEGR(T123) | Rv2890c | rpsB | -0.829187601 | 1.708701856 | 3.827600157 |
| 152 | IVVEFGsVDDLAR(S319) | Rv3917c | parB | -0.770284669 | 1.728158735 | 3.858487577 |
| 153 | GSDtAVIR(T217) | Rv3140 | fadE23 | -1.603789181 | 1.73619787 | 3.029587522 |
| 154 | IsGDLK(S22) | Rv3874 | esxB | -1.179899607 | 1.738041041 | 4.205795113 |
| 155 | DDsGLLTLTDER(S37) | Rv3208A | TB9.4 | -0.367629155 | 1.763623236 | 4.067535612 |
| 156 | LLGSFELtGIPPAPR(T434) | Rv0350 | dnaK | -0.746862275 | 1.764816176 | 4.375093239 |
| 157 | sDLLEANGAIFTAQGK(S100) | Rv1240 | mdh | -0.563632015 | 1.777932434 | 3.956406715 |
| 158 | GEGFDtAVIGDGTQALtAVR(T32; T43) | Rv3246c | mtrA | -0.458985515 | 1.779640098 | 3.953401424 |
| 159 | MQEQVsASLR(S168) | Rv2744c | 35kd_ag | -0.683052361 | 1.794348873 | 3.213985201 |
| 160 | SASsTVEAPVAR(S38) | Rv2839c | infB | -0.781707857 | 1.794644068 | 3.891087846 |
| 161 | YNGEEyLILSAR(Y86) | Rv3418c | groES | -0.467828777 | 1.808435443 | 4.069878504 |
| 162 | MTIVVSAEDTPLEQItK(T64) | Rv3002c | ilvN | -0.038234613 | 1.814961578 | 4.070395858 |
| 163 | AMGtDLLTIR(T119) | Rv1871c | Rv1871c | -0.003403892 | 1.81537693 | 3.907090144 |
| 164 | GYEtAESLAAR(T394) | Rv0873 | fadE10 | -1.742891302 | 1.836203333 | 2.679662635 |
| 165 | GISAAEVVDtR(T37) | Rv2533c | nusB | 0.186876297 | 1.851317294 | 4.196930262 |
| 166 | AAGIDtVVFDR(T94) | Rv0720 | rplR | 0.652225074 | 1.875995634 | 4.742386238 |
| 167 | VEKDPENPtVVLtVR(T213; T217) | Rv3246c | mtrA | -0.377716974 | 1.905060481 | 3.78003582 |
| 168 | VPAENAEyVGVR(Y55) | Rv3583c | Rv3583c | 0.507402316 | 1.918583983 | 5.3549131 |
| 169 | GGGGGDDDDIAGStAAGQER(T22) | Rv2111c | pup | -0.493866233 | 1.931103066 | 3.207298134 |
| 170 | VLVVDDDsDVLASLER(S20) | Rv0903c | prrA | 0.270134808 | 1.932918011 | 4.289791926 |
| 171 | YSSPQtDFQR(T24) | Rv0810c | Rv0810c | -0.091238053 | 1.933401903 | 4.013195774 |
| 172 | RtETDDVSEDSLEELK(T11) | Rv2699c | Rv2699c | -0.023637377 | 1.935551305 | 4.113765432 |
| 173 | GAAGtAAQAAVVR(T49) | Rv3874 | esxB | -0.605908718 | 1.942591513 | 3.520288978 |
| 174 | APIDAGtAASQR(T390) | Rv3864 | espE | -0.383588623 | 1.969132857 | 3.248861998 |
| 175 | YIsQIDVER(S71) | Rv2785c | rpsO | 0.369992257 | 2.00380664 | 5.262952788 |
| 176 | GGESGSPSTsLVLDQFGR(S168) | Rv3596c | clpC1 | -0.414734341 | 2.014778967 | 4.550511827 |
| 177 | VEKDPENPtVVLTVR(T213) | Rv3246c | mtrA | -0.34862871 | 2.023197267 | 3.451970087 |
| 178 | VEKDPENPtVVLTVR(T213) | Rv3246c | mtrA | -0.401969431 | 2.024920525 | 3.568984191 |
| 179 | NQAEtLVYQTEK(T515) | Rv0350 | dnaK | -0.554681408 | 2.030492935 | 3.699565218 |
| 180 | DDtLPMLTGIR(T160) | Rv0002 | dnaN | -0.971359299 | 2.032801654 | 4.029178894 |
| 181 | EKPQEGtVVAVGPGR(T42) | Rv3418c | groES | -0.211843723 | 2.035755718 | 4.095780108 |
| 182 | ADDtDDVILNR(T133) | Rv0733 | adk | 0.077981467 | 2.050792846 | 4.352581827 |
| 183 | QEItEVIALQER(T472) | Rv1133c | metE | -0.847545524 | 2.069290841 | 3.919530729 |
| 184 | MSAPYVEGASIDFVDtIEK(T94) | Rv2204c | Rv2204c | -0.112519994 | 2.071377889 | 4.964192882 |
| 185 | DtLIMSYEWPEDR(T64) | Rv2185c | TB16.3 | 0.510408525 | 2.072729107 | 4.34466739 |
| 186 | IDtSGLPAVGDDAtVPR(T72; T83) | Rv2921c | ftsY | -0.854683681 | 2.073764809 | 3.890397365 |
| 187 | AIEILQtGVIR(T379) | Rv1872c | lldD2 | -0.637574046 | 2.07770709 | 3.21641735 |
| 188 | GSAAPVDsNADAGGFDQFNR(S245) | Rv2145c | wag31 | -0.327612096 | 2.088869626 | 3.766275538 |
| 189 | AIEVtSEEVYNR(T263) | Rv0642c | mmaA4 | -0.707620193 | 2.114260641 | 4.370035011 |
| 190 | sQVIEAVNLFR(S102) | Rv3002c | ilvN | -0.378618817 | 2.134512228 | 4.340109032 |
| 191 | TTESDtPtEVIR(T207; T209) | Rv2536 | Rv2536 | -0.902627868 | 2.168895928 | 3.782859889 |
| 192 | VYEGAAPHLtPVtLELGGK(T249; T252) | Rv0147 | Rv0147 | -1.391095263 | 2.186498602 | 3.479706284 |
| 193 | ILPVDFTEDTGELtPTMK(T575) | Rv2187 | fadD15 | -1.605116046 | 2.191965948 | 3.319614842 |
| 194 | ASDVAsDIGSFIR(S20) | Rv0474 | Rv0474 | -0.954443296 | 2.216149937 | 4.537698774 |
| 195 | IQFTPDLVPtDIIGTR(T111) | Rv1479 | moxR1 | -0.671986278 | 2.244989383 | 4.580888656 |
| 196 | LQEDLPEQLtELR(T69) | Rv0475 | hbhA | -0.378819773 | 2.249881927 | 5.126679557 |
| 197 | VVFINtGFLDR(T458) | Rv1837c | glcB | -0.891411927 | 2.271490876 | 4.501236871 |
| 198 | GDEAVELTSStEER(T55) | Rv0566c | Rv0566c | 0.163809591 | 2.280679484 | 5.366862652 |
| 199 | VtDEFAALRPPGQL(T224) | Rv1593c | Rv1593c | 0.086588155 | 2.282970852 | 4.05080224 |
| 200 | TDTEADQtKPGDEPK(T221) | Rv2536 | Rv2536 | -0.787447396 | 2.285918876 | 3.127913363 |
| 201 | FDPEDtSEQLVIVGER(T552) | Rv3801c | fadD32 | -0.797051649 | 2.293729367 | 4.061463637 |
| 202 | LEGDEAtGANIVK(T435) | Rv0440 | groEL2 | -0.447783636 | 2.302897574 | 4.100434346 |

| | | | | | | |
|---|---|---|---|---|---|---|
| 203 | sVNEGFSGGEK(S146) | Rv1463 | Rv1463 | -0.498318887 | 2.305680734 | 4.363918705 |
| 204 | GEtQILGVTTLDMIK(T379) | Rv2783c | gpsI | -1.016496986 | 2.321573057 | 3.730760588 |
| 205 | QATAAVENVVDtIVR(T31) | Rv2986c | hupB | -0.405616624 | 2.321681957 | 4.815872408 |
| 206 | EDQsDLIYK(S405) | Rv3240c | secA1 | -0.863076495 | 2.338226751 | 3.483988615 |
| 207 | SAtDIGGLPGK(T433) | Rv0005 | gyrB | -0.634938612 | 2.342128654 | 4.239480602 |
| 208 | EDFtGEGAK(T279) | Rv0824c | desA1 | -0.929337562 | 2.346183581 | 4.978354044 |
| 209 | TNPSGAtMAALANFFR(T60) | Rv3849 | espR | 0.479760291 | 2.349814079 | 5.251827017 |
| 210 | VTIVGLPDIPGyAAK(Y278) | Rv3709c | ask | -1.137995748 | 2.378553226 | 3.901348272 |
| 211 | IEtNGQLAAAAK(T72) | Rv3852 | hns | -0.404936299 | 2.433583116 | 4.271890736 |
| 212 | ADIEAtEVR(T121) | Rv3105c | prfB | -0.790871836 | 2.44826569 | 3.849023243 |
| 213 | QEIDQtRDQLAAtIDSLAER(T16; T23) | Rv2520c | Rv2520c | -1.577936028 | 2.461460628 | 4.038957589 |
| 214 | WDATAtELNNALQNLAR(T63) | Rv3875 | esxA | -0.312455507 | 2.463830781 | 4.421358041 |
| 215 | GMSGLEDsSDLVVSPYVR(S11) | Rv3411c | guaB2 | -0.369053709 | 2.474674239 | 4.486618929 |
| 216 | SIGDVPtGIDLTK(T205) | Rv0284 | eccC3 | -1.198171795 | 2.520388336 | 4.235091433 |
| 217 | TLDGDQtAEFGGVR(T66) | Rv2204c | Rv2204c | -0.168118544 | 2.523888566 | 4.538610964 |
| 218 | ELSGtGTDR(T36) | Rv0810c | Rv0810c | 0.577038092 | 2.525765224 | 4.018265135 |
| 219 | SFtFTLK(T69) | Rv0640 | rplK | 0.08840697 | 2.533213201 | 5.118048625 |
| 220 | ATDtVAGISGR(T32) | Rv3865 | espF | 0.252279772 | 2.539387007 | 4.311749685 |
| 221 | TTVtGVEMFR(T259) | Rv0685 | tuf | -0.470316589 | 2.552236482 | 4.699791373 |
| 222 | QGLtLPASVDLEK(T10\|\|T10) | Rv0814c; Rv3118 | sseC1/sseC2 | -0.147533662 | 2.560009087 | 4.180965913 |
| 223 | tLVELFGLAR(T214) | Rv3457c | rpoA | -0.940139665 | 2.587039923 | 4.271346011 |
| 224 | EyLVLK(Y34) | Rv3583c | Rv3583c | 0.098249482 | 2.589070107 | 5.716742256 |
| 225 | ILPVDFTEDtGELtPTMK(T571; T575) | Rv2187 | fadD15 | -1.496917534 | 2.598787617 | 3.924463871 |
| 226 | FALYSVSDTPETTtASR(T126) | Rv1109c | Rv1109c | -0.393179532 | 2.668411975 | 4.60056743 |
| 227 | VTGtAPIYEILHDAK(T63) | Rv1636 | TB15.3 | 0.220437439 | 2.717643224 | 5.116990531 |
| 228 | VGDyLVVK(Y8) | Rv2302 | Rv2302 | 1.340375184 | 2.737772037 | 5.433676464 |
| 229 | SEALAASVLPDRtI(T219) | Rv0721 | rpsE | -1.020710783 | 2.759472854 | 4.16495813 |
| 230 | TGDGPLEAtKEGR(T36) | Rv1211 | Rv1211 | 1.485542809 | 2.775027813 | 4.798811845 |
| 231 | IITAAtVVLPFK(T148) | Rv2987c | leuD | -0.072891585 | 2.778633376 | 5.831417215 |
| 232 | SNVDtDQIIPAVFLK(T21) | Rv2987c | leuD | 0.018788459 | 2.84626003 | 5.657967713 |
| 233 | LStDELLDAFK(T6) | Rv0652 | rplL | -0.045643972 | 2.848157103 | 5.911545273 |
| 234 | EEESAQtFK(T448) | Rv3801c | fadD32 | -0.968433966 | 2.878245465 | 4.723868475 |
| 235 | VLPGyILVR(Y108) | Rv0639 | nusG | -0.389443455 | 2.879623955 | 5.080970145 |
| 236 | VQNLDVGDyIFQVEVPTEEVTEIK(Y78) | Rv0639 | nusG | -0.593666068 | 2.894322281 | 6.062909401 |
| 237 | AAVEAATVTDSDGNTIDtSEFFGK(T433) | Rv2462c | tig | -0.344638145 | 2.960373026 | 4.906617137 |
| 238 | TGDGPLEAtKEGR(T36) | Rv1211 | Rv1211 | 1.158440395 | 2.98583995 | 4.658880144 |
| 239 | VSLPVALVVDGATVTtDAK(T51) | Rv2442c | rplU | 0.187576914 | 3.034310226 | 5.565858746 |
| 240 | SSALPtGVEALK(T35) | Rv0871 | cspB | 0.310995585 | 3.065142263 | 5.304708092 |
| 241 | RAtIEQLLtIPLAK(T574; T580) | Rv1837c | glcB | -1.171838244 | 3.190342769 | 5.05312575 |
| 242 | SLLTSGAtDAK(T91) | Rv2229c | Rv2229c | -0.843582665 | 3.218690336 | 4.735883642 |
| 243 | EALIEsGVLIPAR(S61) | Rv3407 | vapB47 | 0.617388384 | 3.253614729 | 5.421655897 |
| 244 | GDIAGGAsGIVK(S43) | Rv1893 | Rv1893 | -0.529847767 | 3.330527066 | 4.284178562 |
| 245 | tAVLDFIDR(T261) | Rv1021 | Rv1021 | -0.146254229 | 3.330749402 | 5.051152256 |
| 246 | FQPVQVGEPtVEHtIEILK(T351; T355) | Rv3596c | clpC1 | -0.497065226 | 3.401792852 | 5.699897705 |
| 247 | FPDLNEtK(T46) | Rv0685 | tuf | -1.414248666 | 3.503299507 | 4.11474521 |
| 248 | TDtEADQtKPGDEPK(T216; T221) | Rv2536 | Rv2536 | -1.310748766 | 3.511437328 | 3.896884188 |
| 249 | RIDPEtGEVR(T28) | Rv0351 | grpE | -0.164957076 | 3.553170392 | 5.238043299 |
| 250 | VSItDAAMVAAATLADR(T375) | Rv3596c | clpC1 | -0.327526765 | 3.564474146 | 4.592834618 |
| 251 | LSFEtAR(T197) | Rv0642c | mmaA4 | -0.83782043 | 3.705731793 | 5.101605386 |
| 252 | GGtNLtQVLK(T122; T125) | Rv1388 | mihF | 0.556427526 | 3.887036801 | 5.965092623 |
| 253 | FAsDIEAIYNKE(S591) | Rv2187 | fadD15 | -1.144891307 | 3.915359854 | 4.973360155 |
| 254 | EMtLLELSDFVK(T17) | Rv0652 | rplL | -0.392286949 | 3.92682423 | 6.281701218 |
| 255 | IPLDVAEGDtVIYSK(T68) | Rv3418c | groES | -0.264671484 | 3.930961958 | 5.595889198 |

| | | | | | | |
|---|---|---|---|---|---|---|
| 256 | FPDLNEtK(T46) | Rv0685 | tuf | -0.683983436 | 3.964961019 | 5.424689241 |
| 257 | EDVtEIILNLK(T74) | Rv3457c | rpoA | -0.806430966 | 4.383399494 | 5.809830179 |

| Supplementary Table 3a: Mean normalized log2 intenstity ratios of phosphopeptides in which the phosphorylation is dependent on PknB. The values were used for the generation of heat map in Figure 7c. | | | | | | |
|---|---|---|---|---|---|---|
| **S.No** | **Phosphopeptides (Phosphosite)** | **Gene Number** | **Gene Name** | **Depletion (Log2)** | **3F-PknB WT (Log2)** | **3F-PknB GM (Log2)** |
| 1 | AAVPQPsEK(S69) | Rv0250c | Rv0250c | -1.213708729 | 1.007787147 | 2.324867142 |
| 2 | ILDsGGR(S54) | Rv1298 | rpmE | -0.346072302 | 1.028754061 | 4.198785482 |
| 3 | DLtEEQLIHLR(T49) | Rv3460c | rpsM | -0.323282689 | 1.029370213 | 3.778102199 |
| 4 | tGIAQVATVSSR(T141) | Rv3417c | groEL1 | -0.859984901 | 1.029673006 | 2.81684682 |
| 5 | GSLDtQGVK(T104) | Rv0682 | rpsL | -0.497073958 | 1.072662397 | 3.24750367 |
| 6 | tLDQIGK(T283) | Rv2710 | sigB | -1.36358639 | 1.077323404 | 3.152646176 |
| 7 | GVLDEAtGR(T153) | Rv1488 | Rv1488 | -1.279929335 | 1.100184529 | 2.495272355 |
| 8 | GVGyVLR(Y241) | Rv0757 | phoP | -0.944935927 | 1.102275188 | 3.315588599 |
| 9 | QDAtVEVAIR(T41) | Rv0641 | rplA | -0.65102661 | 1.135069139 | 3.231507882 |
| 10 | VDAtRVEQR(T181) | Rv3457c | rpoA | -0.86103971 | 1.139325461 | 2.872109466 |
| 11 | NQAVtNVDR(T60) | Rv0350 | dnaK | -0.975436072 | 1.140299946 | 3.073934409 |
| 12 | DQPFItGER(T250) | Rv0467 | icl1 | -2.022854105 | 1.140573584 | 1.756721344 |
| 13 | AAAyLQAQGQAK(Y247) | Rv1488 | Rv1488 | -1.527184053 | 1.175546823 | 2.801227999 |
| 14 | MDLtDDSWAAVR(T116) | Rv0639 | nusG | -0.778437341 | 1.193308075 | 3.369012285 |
| 15 | IAGsQVLK(S594) | Rv0684 | fusA1 | -1.274963436 | 1.194673513 | 2.556237073 |
| 16 | LAADVADVPtVVIAR(T223) | Rv0467 | icl1 | -1.78998422 | 1.206168039 | 2.332763078 |
| 17 | AGILEPSETsQVR(S93) | Rv0474 | Rv0474 | -1.419921407 | 1.23382812 | 3.15770163 |
| 18 | DAITDtAItER(T103; T106) | Rv0474 | Rv0474 | -1.342737486 | 1.2602227 | 3.404464861 |
| 19 | SETFTTADDNQPsVQIQVYQGER(S409) | Rv0350 | dnaK | -0.640084525 | 1.298182346 | 3.518799337 |
| 20 | VtETLLK(T127) | Rv0440 | groEL2 | -0.705459387 | 1.32874573 | 3.195851889 |
| 21 | FTSEAALFGNDLAtQPNYPAEIR(T61) | Rv2455c | Rv2455c | -0.785455517 | 1.331770556 | 3.262073385 |
| 22 | DETTIVEGAGDtDAIAGR(T337) | Rv0440 | groEL2 | -0.399621187 | 1.345267326 | 3.039975608 |
| 23 | TAMsAVDDLR(S212) | Rv1265 | Rv1265 | -0.894044774 | 1.372148977 | 2.462711336 |
| 24 | AMILTAEGtR(T198) | Rv1488 | Rv1488 | -1.401960306 | 1.413327479 | 2.778004428 |
| 25 | YPHQPAPtGA(T264) | Rv1463 | Rv1463 | -0.39701753 | 1.451884901 | 3.476299366 |
| 26 | TLLSSIPGAAVtSIR(T52) | Rv3457c | rpoA | -1.122206701 | 1.45526077 | 3.496764417 |
| 27 | VPGyAPQGGGYAEPAGR(Y388) | Rv0020c | fhaA | -0.400784793 | 1.477677464 | 3.678650319 |
| 28 | QEIDQtRDQLAATIDSLAER(T16) | Rv2520c | Rv2520c | -1.753158453 | 1.491505891 | 2.977023547 |
| 29 | QYAEyVK(Y31) | Rv0707 | rpsC | -1.686252284 | 1.503469726 | 2.736413967 |
| 30 | AEAsIETPtPVQSQR(S55; T60) | Rv2094c | tatA | -0.370095698 | 1.509749048 | 3.276080188 |
| 31 | ETPVELtFGQVSK(T231) | Rv0639 | nusG | -0.662080756 | 1.524168167 | 4.567149698 |
| 32 | FGLtDGQPR(T482) | Rv2703 | sigA | -0.82717448 | 1.538848672 | 3.859620619 |

| | | | | | | |
|---|---|---|---|---|---|---|
| 33 | FtDSQLR(T217) | Rv1593c | Rv1593c | -1.162025401 | 1.575317226 | 3.82503211 |
| 34 | DDtAVLVTLTAR(T226) | Rv3200c | Rv3200c | -0.811683875 | 1.585498873 | 2.915973906 |
| 35 | QTGGsGQFAK(S509) | Rv0684 | fusA1 | -0.596567541 | 1.602668097 | 3.279774122 |
| 36 | YTAPEIsAR(S92) | Rv0350 | dnaK | -0.565287217 | 1.632318523 | 4.368230612 |
| 37 | DQLAAtIDSLAER(T23) | Rv2520c | Rv2520c | -1.410779085 | 1.636891802 | 3.102693316 |
| 38 | TDAAtLAQEAGNFER(T10) | Rv3874 | esxB | -0.558434225 | 1.651325265 | 3.373335182 |
| 39 | YGGtEIK(T77) | Rv3418c | groES | -1.041668572 | 1.661610299 | 3.554351376 |
| 40 | tAIVEGLAQR(T213) | Rv0384c | clpB | -0.714246302 | 1.680239022 | 4.071438864 |
| 41 | VTVtAADLR(T48) | Rv0300 | vapB2 | -0.529912684 | 1.68540779 | 3.793759183 |
| 42 | ELEAMEQtGGFEGR(T123) | Rv2890c | rpsB | -0.829187601 | 1.708701856 | 3.827600157 |
| 43 | IVVEFGsVDDLAR(S319) | Rv3917c | parB | -0.770284669 | 1.728158735 | 3.858487577 |
| 44 | GSDtAVIR(T217) | Rv3140 | fadE23 | -1.603789181 | 1.73619787 | 3.029587522 |
| 45 | IsGDLK(S22) | Rv3874 | esxB | -1.179899607 | 1.738041041 | 4.205795113 |
| 46 | DDsGLLTLTDER(S37) | Rv3208A | TB9.4 | -0.367629155 | 1.763623236 | 4.067535612 |
| 47 | LLGSFELtGIPPAPR(T434) | Rv0350 | dnaK | -0.746862275 | 1.764816176 | 4.375093239 |
| 48 | sDLLEANGAIFTAQGK(S100) | Rv1240 | mdh | -0.563632015 | 1.777932434 | 3.956406715 |
| 49 | GEGFDtAVIGDGTQALtAVR(T32; T43) | Rv3246c | mtrA | -0.458985515 | 1.779640098 | 3.953401424 |
| 50 | MQEQVsASLR(S168) | Rv2744c | 35kd_ag | -0.683052361 | 1.794348873 | 3.213985201 |
| 51 | SASsTVEAPVAR(S38) | Rv2839c | infB | -0.781707857 | 1.794644068 | 3.891087846 |
| 52 | YNGEEyLILSAR(Y86) | Rv3418c | groES | -0.467828777 | 1.808435443 | 4.069878504 |
| 53 | GYEtAESLAAR(T394) | Rv0873 | fadE10 | -1.742891302 | 1.836203333 | 2.679662635 |
| 54 | DPENPtVVLtVR(T213; T217) | Rv3246c | mtrA | -0.377716974 | 1.905060481 | 3.78003582 |
| 55 | GGGGGDDDDIAGStAAGQER(T22) | Rv2111c | pup | -0.493866233 | 1.931103066 | 3.207298134 |
| 56 | GAAGtAAQAAVVR(T49) | Rv3874 | esxB | -0.605908718 | 1.942591513 | 3.520288978 |
| 57 | APIDAGtAASQR(T390) | Rv3864 | espE | -0.383588623 | 1.969132857 | 3.248861998 |
| 58 | GGESGSPSTsLVLDQFGR(S168) | Rv3596c | clpC1 | -0.414734341 | 2.014778967 | 4.550511827 |
| 59 | VEKDPENPtVVLTVR(T213) | Rv3246c | mtrA | -0.34862871 | 2.023197267 | 3.451970087 |
| 60 | VEKDPENPtVVLTVR(T213) | Rv3246c | mtrA | -0.401969431 | 2.024920525 | 3.568984191 |
| 61 | NQAEtLVYQTEK(T515) | Rv0350 | dnaK | -0.554681408 | 2.030492935 | 3.699565218 |
| 62 | DDtLPMLTGIR(T160) | Rv0002 | dnaN | -0.971359299 | 2.032801654 | 4.029178894 |
| 63 | QEItEVIALQER(T472) | Rv1133c | metE | -0.847545524 | 2.069290841 | 3.919530729 |
| 64 | IDtSGLPAVGDDAtVPR(T72; T83) | Rv2921c | ftsY | -0.854683681 | 2.073764809 | 3.890397365 |
| 65 | AIEILQtGVIR(T379) | Rv1872c | lldD2 | -0.637574046 | 2.07770709 | 3.21641735 |
| 66 | GSAAPVDsNADAGGFDQFNR(S245) | Rv2145c | wag31 | -0.327612096 | 2.088869626 | 3.766275538 |
| 67 | AIEVtSEEVYNR(T263) | Rv0642c | mmaA4 | -0.707620193 | 2.114260641 | 4.370035011 |
| 68 | sQVIEAVNLFR(S102) | Rv3002c | ilvN | -0.378618817 | 2.134512228 | 4.340109032 |
| 69 | TTESDtPtEVIR(T207; T209) | Rv2536 | Rv2536 | -0.902627868 | 2.168895928 | 3.782859889 |

| | | | | | | |
|---|---|---|---|---|---|---|
| 70 | VYEGAAPHLtPVtLELGGK(T249; T252) | Rv0147 | Rv0147 | -1.391095263 | 2.186498602 | 3.479706284 |
| 71 | ILPVDFTEDTGELtPTMK(T575) | Rv2187 | fadD15 | -1.605116046 | 2.191965948 | 3.319614842 |
| 72 | ASDVAsDIGSFIR(S20) | Rv0474 | Rv0474 | -0.954443296 | 2.216149937 | 4.537698774 |
| 73 | IQFTPDLVPtDIIGTR(T111) | Rv1479 | moxR1 | -0.671986278 | 2.244989383 | 4.580888656 |
| 74 | LQEDLPEQLtELR(T69) | Rv0475 | hbhA | -0.378819773 | 2.249881927 | 5.126679557 |
| 75 | VVFINtGFLDR(T458) | Rv1837c | glcB | -0.891411927 | 2.271490876 | 4.501236871 |
| 76 | TDTEADQtKPGDEPK(T221) | Rv2536 | Rv2536 | -0.787447396 | 2.285918876 | 3.127913363 |
| 77 | FDPEDtSEQLVIVGER(T552) | Rv3801c | fadD32 | -0.797051649 | 2.293729367 | 4.061463637 |
| 78 | LEGDEAtGANIVK(T435) | Rv0440 | groEL2 | -0.447783636 | 2.302897574 | 4.100434346 |
| 79 | sVNEGFSGGEK(S146) | Rv1463 | Rv1463 | -0.498318887 | 2.305680734 | 4.363918705 |
| 80 | GEtQILGVTTLDMIK(T379) | Rv2783c | gpsI | -1.016496986 | 2.321573057 | 3.730760588 |
| 81 | QATAAVENVVDtIVR(T31) | Rv2986c | hupB | -0.405616624 | 2.321681957 | 4.815872408 |
| 82 | EDQsDLIYK(S405) | Rv3240c | secA1 | -0.863076495 | 2.338226751 | 3.483988615 |
| 83 | SAtDIGGLPGK(T433) | Rv0005 | gyrB | -0.634938612 | 2.342128654 | 4.239480602 |
| 84 | EDFtGEGAK(T279) | Rv0824c | desA1 | -0.929337562 | 2.346183581 | 4.978354044 |
| 85 | VTIVGLPDIPGyAAK(Y278) | Rv3709c | ask | -1.137995748 | 2.378553226 | 3.901348272 |
| 86 | IEtNGQLAAAAK(T72) | Rv3852 | hns | -0.404936299 | 2.433583116 | 4.271890736 |
| 87 | ADIEAtEVR(T121) | Rv3105c | prfB | -0.790871836 | 2.44826569 | 3.849023243 |
| 88 | QEIDQtRDQLAAtIDSLAER(T16; T23) | Rv2520c | Rv2520c | -1.577936028 | 2.461460628 | 4.038957589 |
| 89 | GMSGLEDsSDLVVSPYVR(S11) | Rv3411c | guaB2 | -0.369053709 | 2.474674239 | 4.486618929 |
| 90 | SIGDVPtGIDLTK(T205) | Rv0284 | eccC3 | -1.198171795 | 2.520388336 | 4.235091433 |
| 91 | TTVtGVEMFR(T259) | Rv0685 | tuf | -0.470316589 | 2.552236482 | 4.699791373 |
| 92 | tLVELFGLAR(T214) | Rv3457c | rpoA | -0.940139665 | 2.587039923 | 4.271346011 |
| 93 | ILPVDFTEDtGELtPTMK(T571; T575) | Rv2187 | fadD15 | -1.496917534 | 2.598787617 | 3.924463871 |
| 94 | FALYSVSDTPETTtASR(T126) | Rv1109c | Rv1109c | -0.393179532 | 2.668411975 | 4.60056743 |
| 95 | SEALAASVLPDRtI(T219) | Rv0721 | rpsE | -1.020710783 | 2.759472854 | 4.16495813 |
| 96 | EEESAQtFK(T448) | Rv3801c | fadD32 | -0.968433966 | 2.878245465 | 4.723868475 |
| 97 | VLPGyILVR(Y108) | Rv0639 | nusG | -0.389443455 | 2.879623955 | 5.080970145 |
| 98 | VQNLDVGDyIFQVEVPTEEVTEIK(Y78) | Rv0639 | nusG | -0.593666068 | 2.894322281 | 6.062909401 |
| 99 | AAVEAATVTDSDGNTIDtSEFFGK(T433) | Rv2462c | tig | -0.344638145 | 2.960373026 | 4.906617137 |
| 100 | RAtIEQLLtIPLAK(T574; T580) | Rv1837c | glcB | -1.171838244 | 3.190342769 | 5.05312575 |
| 101 | SLLTSGAtDAK(T91) | Rv2229c | Rv2229c | -0.843582665 | 3.218690336 | 4.735883642 |
| 102 | GDIAGGAsGIVK(S43) | Rv1893 | Rv1893 | -0.529847767 | 3.330527066 | 4.284178562 |
| 103 | FQPVQVGEPtVEHtIEILK(T351; T355) | Rv3596c | clpC1 | -0.497065226 | 3.401792852 | 5.699897705 |
| 104 | FPDLNEtK(T46) | Rv0685 | tuf | -1.414248666 | 3.503299507 | 4.11474521 |
| 105 | TDtEADQtKPGDEPK(T216; T221) | Rv2536 | Rv2536 | -1.310748766 | 3.511437328 | 3.896884188 |
| 106 | VSItDAAMVAAATLADR(T375) | Rv3596c | clpC1 | -0.327526765 | 3.564474146 | 4.592834618 |

| | | | | | | |
|---|---|---|---|---|---|---|
| 107 | LSFEtAR(T197) | Rv0642c | mmaA4 | -0.83782043 | 3.705731793 | 5.101605386 |
| 108 | FAsDIEAIYNKE(S591) | Rv2187 | fadD15 | -1.144891307 | 3.915359854 | 4.973360155 |
| 109 | EMtLLELSDFVK(T17) | Rv0652 | rplL | -0.392286949 | 3.92682423 | 6.281701218 |
| 110 | FPDLNEtK(T46) | Rv0685 | tuf | -0.683983436 | 3.964961019 | 5.424689241 |
| 111 | EDVtEIILNLK(T74) | Rv3457c | rpoA | -0.806430966 | 4.383399494 | 5.809830179 |

**Supplementary Table 3b: Substrates of PknB that are common with Carrette et al.**

| S.No | Tuberculist ID | Protein name |
|---|---|---|
| 1 | Rv0020c | FhaA |
| 2 | Rv1298 | RpmE |
| 3 | Rv2094c | TatA |
| 4 | Rv2187 | FadD15 |
| 5 | Rv2536 | Rv2536 |
| 6 | Rv2921c | FtsY |
| 7 | Rv2986c | HupB |
| 8 | Rv3246c | MtrA |
| 9 | Rv3874 | EsxB |

**Supplementary Table 4a: Tyrosine phosphorylated peptides found in the study.**

| S.No | Phosphopeptides (Phosphosites) | Gene number | Gene name | Depletion (Log2) | 3F-PknB WT (Log2) | 3F-PknB GM (Log2) |
|---|---|---|---|---|---|---|
| 1 | LSGyLAAAK(Y337) | Rv0147 | *Rv0147* | -2.18057 | 0.951663 | 2.278460277 |
| 2 | AQyLSGR(Y612) | Rv1925 | *fadD31* | -2.16342 | 0.788185 | 1.48406619 |
| 3 | VyNTAEQNAANAGK(Y284) | Rv2187 | *fadD15* | -1.17784 | 0.524482 | 1.789250326 |
| 4 | TyLESQLEELGQR(Y226) | Rv2145c | *wag31* | -0.83465 | 0.709687 | 2.761180938 |
| 5 | TIAyDEEAR(Y7) | Rv0440 | *groEL2* | -0.81826 | -0.23838 | 1.563176256 |
| 6 | GGYPPETGGYPPQPGyPRPR(Y215) | Rv0020c | *fhaA* | -0.18941 | -0.49417 | 1.175654461 |
| 7 | DTTyLYR(Y72) | Rv2534c | *efp* | -0.17554 | 0.643915 | 2.717045928 |
| 8 | DyIEANLK(Y59) | Rv3460c | *rpsM* | -0.11435 | 1.071202 | 4.094828363 |
| 9 | AAEGyLEAATSR(Y87) | Rv0475 | *hbhA* | 0.002059 | 0.91322 | 3.669092829 |
| 10 | ESyGVGVER(Y65) | Rv2904c | *rplS* | 0.077033 | 0.010461 | 2.507469029 |
| 11 | EyLVLK(Y34) | Rv3583c | *Rv3583c* | 0.098249 | 2.58907 | 5.716742256 |
| 12 | VPAENAEyVGVR(Y55) | Rv3583c | *Rv3583c* | 0.507402 | 1.918584 | 5.3549131 |
| 13 | VGDyLVVK(Y8) | Rv2302 | *Rv2302* | 1.340375 | 2.737772 | 5.433676464 |
| 14 | VVVELSPyDLSR(Y61) | Rv3462c | infA | -0.245993181 | 1.519471275 | 3.947650974 |
| 15 | NEGyISDFR(Y45) | Rv0718 | rpsH | -0.269410163 | 1.648133991 | 4.602312264 |

**Supplementary Table 4b: Tyrosine phosphorylated peptides wherein the phosphoryaltion is dependent on PknB. The data was used for generating Figure 7d.**

| S.No | Phosphopeptides (Phosphosites) | Gene number | Gene name | Depletion (Log2) | 3F-PknB WT (Log2) | 3F-PknB GM (Log2) |
|---|---|---|---|---|---|---|
| 1 | GVGyVLR(Y241) | Rv0757 | *phoP* | -0.94494 | 1.102275 | 3.315588599 |
| 2 | AAAyLQAQGQAK(Y247) | Rv1488 | *Rv1488* | -1.52718 | 1.175547 | 2.801227999 |
| 3 | VPGyAPQGGGYAEPAGR(Y388) | Rv0020c | *fhaA* | -0.40078 | 1.477677 | 3.678650319 |
| 4 | QYAEyVK(Y31) | Rv0707 | *rpsC* | -1.68625 | 1.50347 | 2.736413967 |
| 5 | YNGEEyLILSAR(Y86) | Rv3418c | *groES* | -0.46783 | 1.808435 | 4.069878504 |
| 6 | VTIVGLPDIPGyAAK(Y278) | Rv3709c | *ask* | -1.138 | 2.378553 | 3.901348272 |
| 7 | VLPGyILVR(Y108) | Rv0639 | *nusG* | -0.38944 | 2.879624 | 5.080970145 |
| 8 | VQNLDVGDyIFQVEVPTEEVTEIK(Y78) | Rv0639 | *nusG* | -0.59367 | 2.894322 | 6.062909401 |

**Supplemenary Table 4c: Data of different clusters used for generation of of Figure 7e**

**Cluster 1**

| S.No | Tuberculist ID | Protein name with phosphosite | Depletion (Log2) | 3F-PknB WT (Log2) | 3F-PknB GM (Log2) |
|---|---|---|---|---|---|
| 1 | Rv0251c | Hsp(T150) | -1.028545672 | -2.073393844 | -1.88049 |
| 2 | Rv0020c | FhaA(T116) | -0.389332853 | -1.83625844 | -0.81065 |
| 3 | Rv1827 | GarA(T21) | -0.378167452 | -1.301596482 | -0.54739 |
| 4 | Rv2536 | Rv2536(T173) | -0.668678032 | -1.109133129 | -0.15862 |
| 5 | Rv2921c | FtsY(S73) | -1.362618269 | -1.799975989 | -0.43192 |

**Cluster 2**

| S.No | Tuberculist ID | Protein name with phosphosite | Depletion (Log2) | 3F-PknB WT (Log2) | 3F-PknB GM (Log2) |
|---|---|---|---|---|---|
| 1 | Rv0639 | NusG(T116) | -0.778437341 | 1.1933081 | 3.369012285 |
| 2 | Rv2710 | SigB(T283) | -1.36358639 | 1.0773234 | 3.152646176 |
| 3 | Rv3240c | SecA1(S405) | -0.863076495 | 2.3382268 | 3.483988615 |

| | | | | | |
|---|---|---|---|---|---|
| 4 | Rv0467 | Icl1(T223) | -1.78998422 | 1.206168 | 2.332763078 |
| 5 | Rv2094c | tatA(S55,T60) | -0.370095698 | 1.509749048 | 3.276080188 |
| **Cluster 3** | | | | | |
| **S.No** | **Tuberculist ID** | **Protein name with phosphosite** | **Depletion (Log2)** | **3F-PknB WT (Log2)** | **3F-PknB GM (Log2)** |
| 1 | Rv2442c | RplU(T62) | 0.033142166 | 0.192658741 | 3.13408628 |
| 2 | Rv1626 | Rv1626(S146) | 0.129994517 | 0.581474618 | 3.058565323 |
| 3 | Rv2986c | HupB(S90) | 0.158295771 | -0.423657923 | 2.775386017 |
| 4 | Rv2050 | Rv2050(T18) | 0.087969287 | 0.144908211 | 2.050186025 |
| 5 | Rv0020c | FhaA(S196) | 0.173189655 | 0.571572675 | 2.142939822 |

Supplementary Figure 1

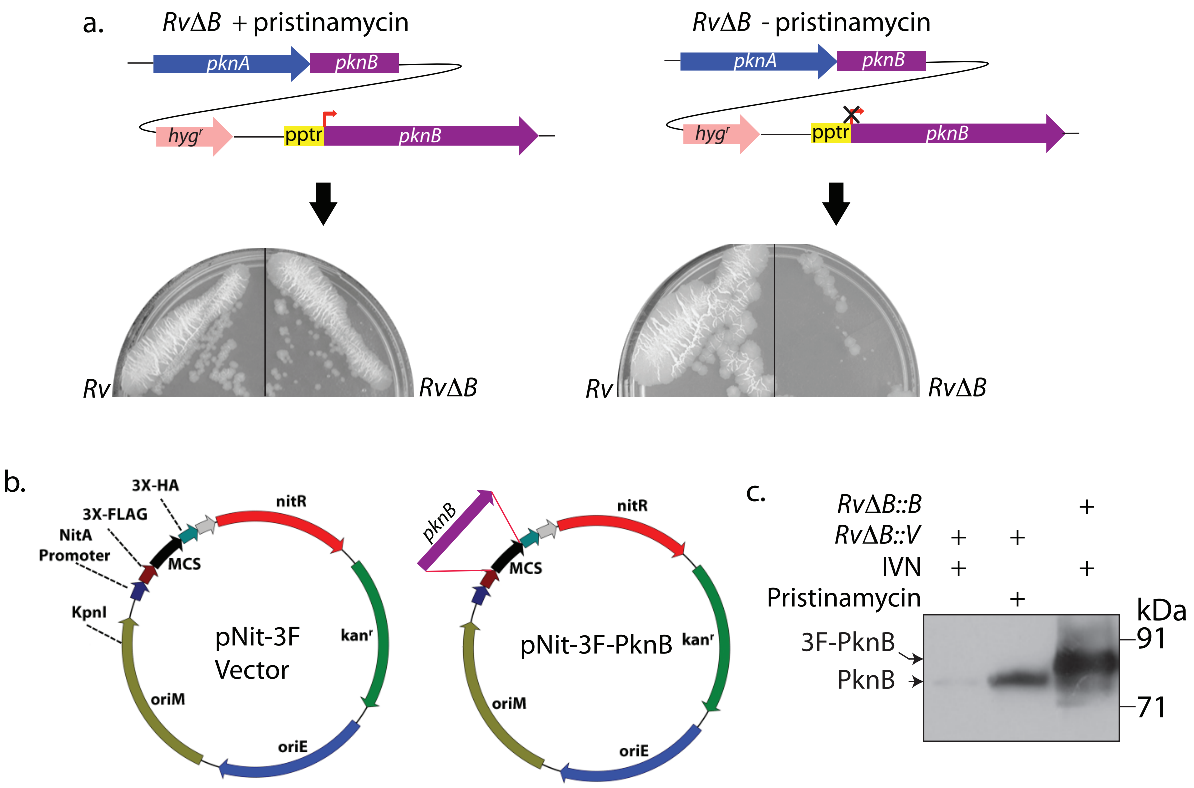


Supplementary Figure1. Schematic representation of the H37Rv conditional PknB knockdown and complementation system used in the study. a. Upper panel: Schematic representation of the modified gene locus in the conditional pknB mutant strain RvΔB. Expression of pknB is under the regulation of pristinamycin inducible promoter pptr. In the 7H9 plates containing pristinamycin (left), both Rv and RvΔB show robust growth. On the contrary RvΔB fails to grow on 7H9 plates that do not contain pristinamycin (right). b. Nitrile inducible episomal expression vector pNit1 was modified to include a N-terminal 3X FLAG tag (left panel) and pknB gene was cloned in the MCS to generate pNit-3F-PknB (right panel). c. RvΔB was transformed with pNit-3F-PknB to generate RvΔB::B strain. RvΔB & RvΔB::B strains were grown in the presence or absence of 1.5 μg/ml pristinamycin or 0.2 μM isovaleronitrile (IVN) as indicated for six days and the WCLs were probed with α-PknB antibodies. Results show that in the absence of pristinamycin, endogenous PknB is very effectively depleted (compare lane 2 with 1). 3F-PknB expressed from the episomal pNit-3F-PknB construct is larger by 3 kDa and hence it migrates slower in the blot (lane 3).

Supplementary Figure 2

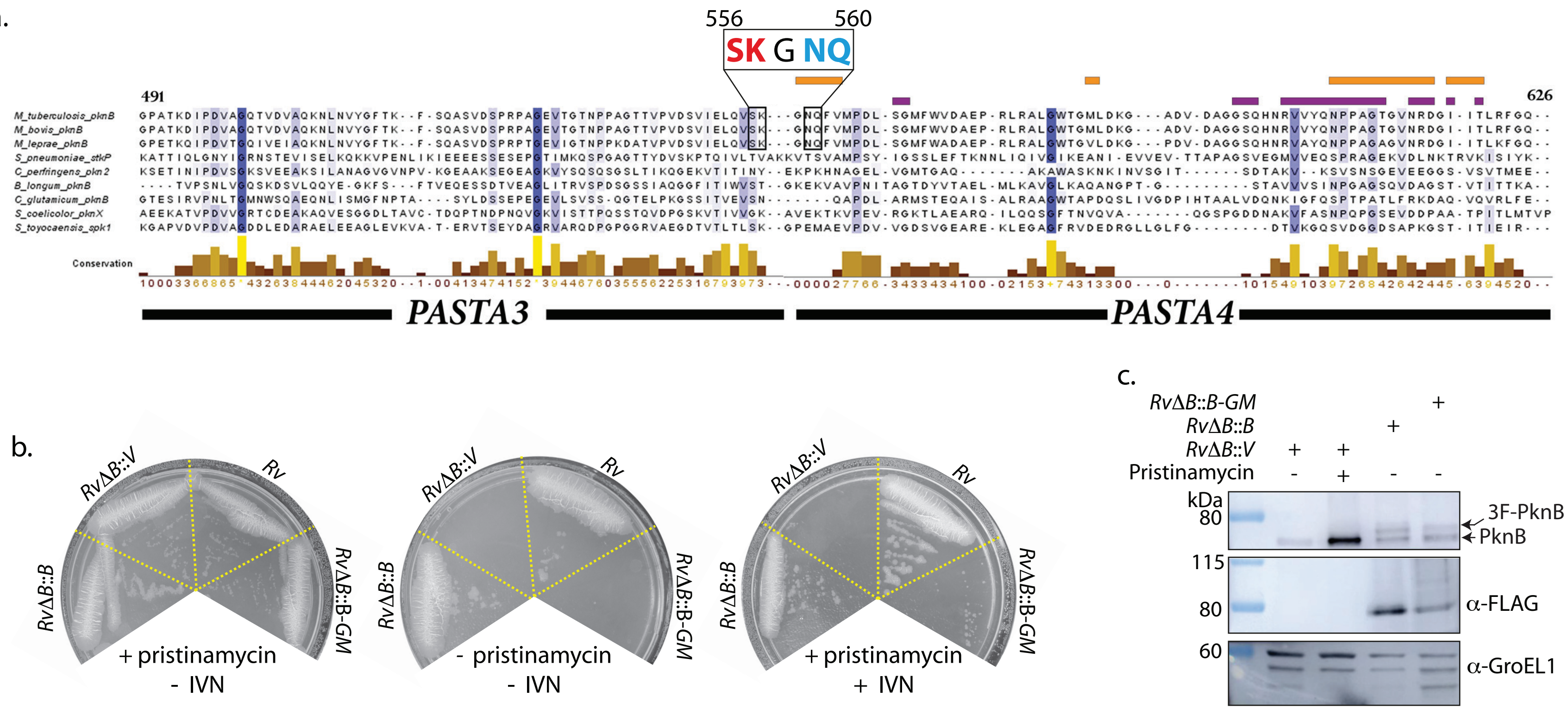


Supplementary Figure2. a. Multiple sequence alignment of PASTA 3 and PASTA 4 domains with the corresponding domains from other prokaryotic PknB orthologs. The domain boundaries of the various PASTA fragments are as per annotation using SWISSPROT, the start and end residue numbering is according to Mtb PknB. The columns are colored as per the degree of conservation of the equivalent residues. Residues forming the PASTA4 dimerization interface are above in purple bars and the residues interacting with the peptidoglycan ligand are marked with orange bars. The crucial residues in the linker region of PASTA3-PASTA4, which exclusively contribute to ligand binding, are marked with boxes. b. RvΔB::V, RvΔB::B & RvΔB::B-GM strains were grown to A600~0.8 in the presence of 1.5 µg/ml pristinamycin. The cultures were washed thrice, diluted to A600~0.05 and streaked on 7H11 plate in the presence or absence of 1.5 µg/ml pristinamycin or 0.2 µM IVN as indicated. c. RvΔB::V, RvΔB::B & RvΔB::B-GM strains cultured to A600~0.8 and were washed thrice with PBST80 to remove pristinamycin. The washed cultures were re-inoculated at A600~0.05 and allowed to grow in presence or absence of pristinamycin for five days. WCLs were prepared and resolved on 10% SDS-PAGE and probed with α-PknB, α-GroEL1 and α-FLAG antibodies.

## Supplementary Figure 3

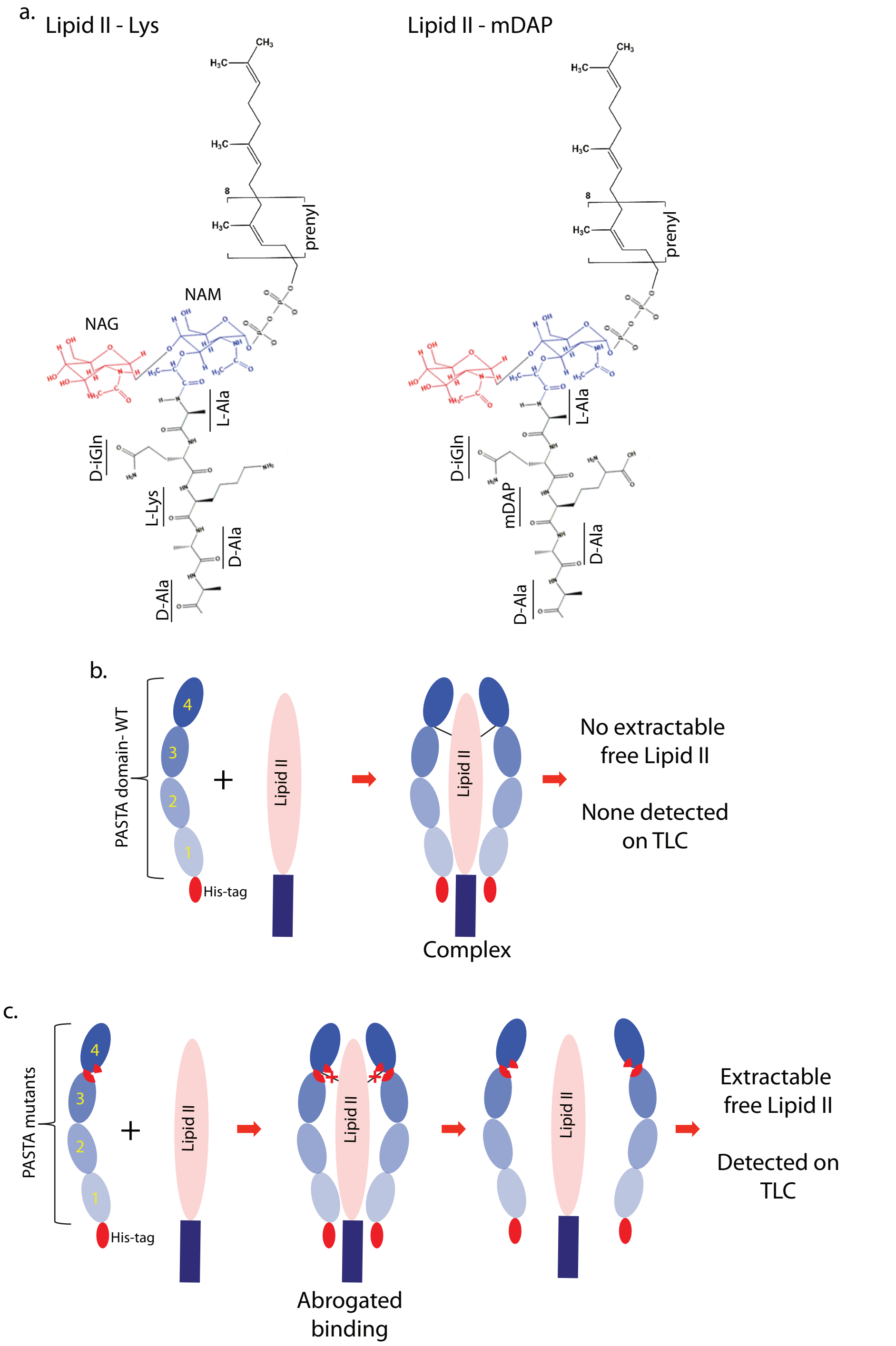


Supplementary Figure3. a. Schematic representation of Lys and mDAP containing LipidII. b & c. Outline of in-vitro LipidII binding assay wherein the ligand was incubated with purified His-PknB-Ec. b. Formation of stable LipidII-PknB complex results in non-availabilty of LipidII for the subsequent organic extraction, thus not detectable on TLC. c. In ability of the His-PknB-Ec mutant proteins to form a complex with LipidII results in successful extraction by organic solvents and subseqeunt detection on TLC.

## Supplementary Figure 4

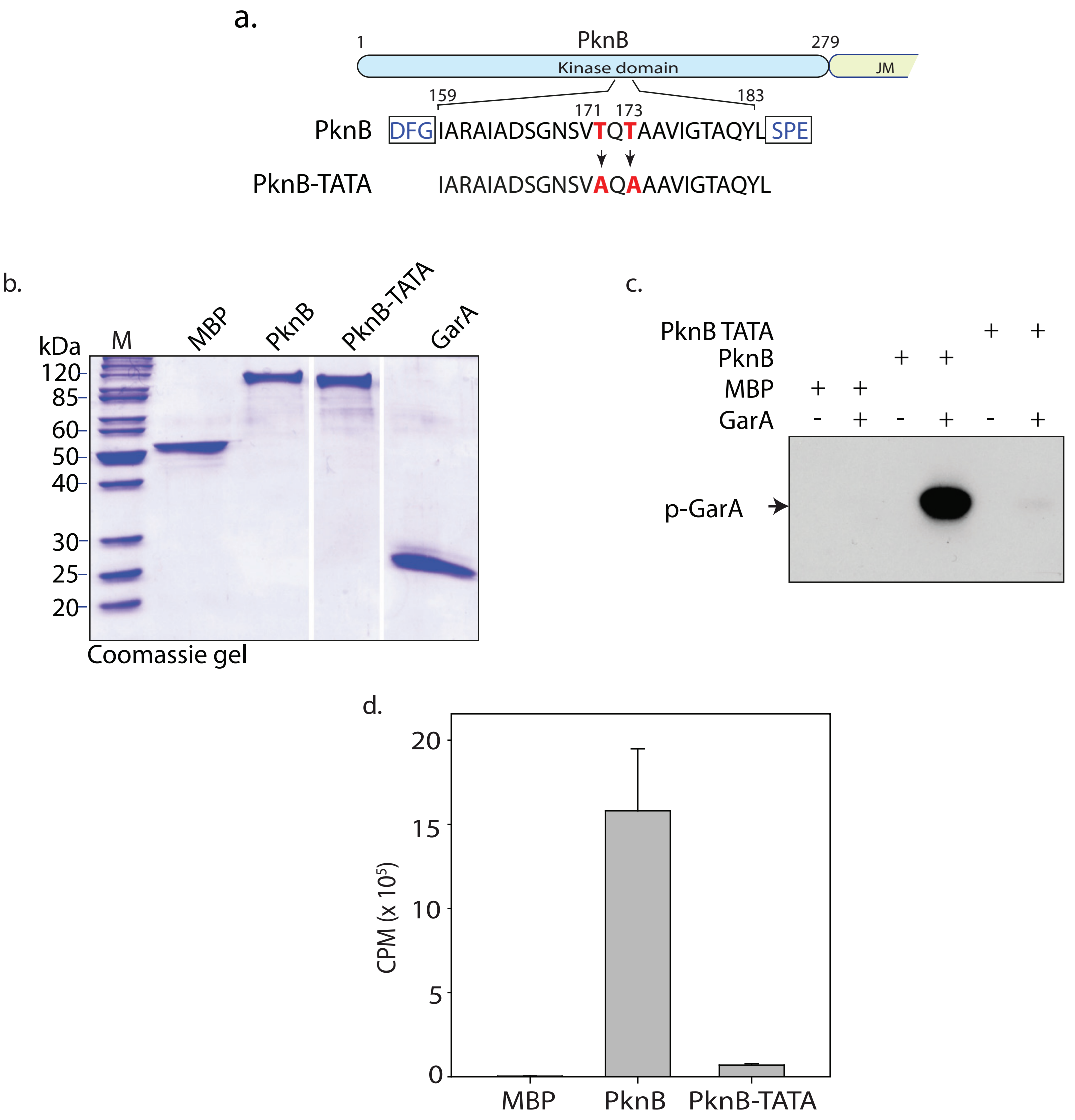


Supplementary Figure 4. Phosphorylation of T171 & T173 in the activation loop is critical for PknB activity. a. Schematic outline showing the activation loop region of PknB. Important activation loop residues are highlighted. T171 and T173 residues in PknB were mutated to alanine residues to generate PknB-TATA. b. The PknB (wild type) and PknB-TATA mutant were subcloned into pMAL-c2X vector. The constructs were transformed into E. coli DE3 Codon plus cells and the MBP (maltose binding protein)-tagged proteins were purified as described 4. 2 μg purified MBP, MBP-PknB, MBP-PknB-TATA and His-GarA were resolved on 12% SDS-PAGE and stained with commassie. c. In vitro kinase assay was performed with 80 nM of MBP, PknB or PknB-TATA in the presence or absence of 3.3 μM GarA in a 30 μl reaction containing 10 μCi [γ32P]ATP and 10 μM ATP. The reactions were resolved on 15% SDS-PAGE and autoradiographed. The reaction was performed in triplicates and the representative autoradiogram is shown. d. Bands corresponding to radiolabelled pGarA were excised and the counts per minute (CPM) were determined using liquid scintillation counter. The activity was calculated as CPM in p-GarA per min per μM enzyme.

Supplementary Figure 5

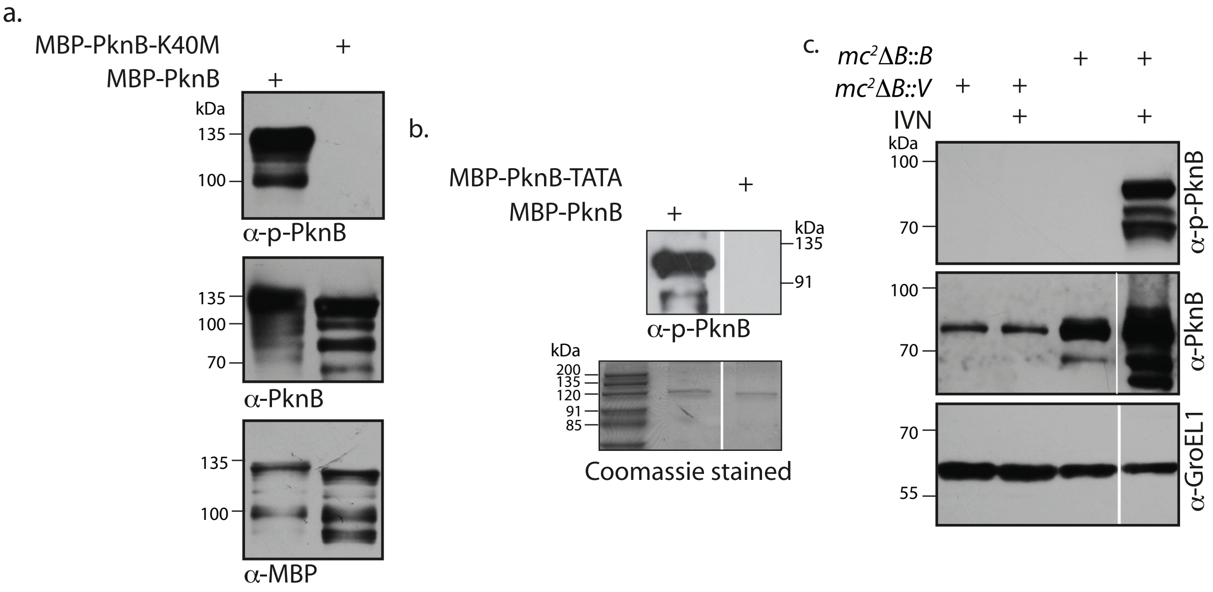


Supplementary Figure5. Characterization of activation loop specific phospho-PknB antibodies a. 100 ng of purified MBP-PknB and MBP-PknB-K40M were resolved, transferred to nitrocellulose and probed with rabbit polyclonal α-pPknB antibody. 10 ng of purified proteins were used for probing with α-PknB and α-MBP antibodies. b. 100 ng of purified of MBP-PknB and MBP-PknB-TATA were resolved and probed with α-pPknB antibody. 20 ng of purified proteins was resolved and strained with coomassie to demonstrate purity and equal concentrations. c. mc2ΔB strain was electroporated with pNit1 or pNit-PknB and transformants were grown in the presence or absence of 5 μM IVN. WCLs were resolved and probed with α-pPknB, α-PknB and α-GroEL1 antibodies.

Suplementary Figure 6

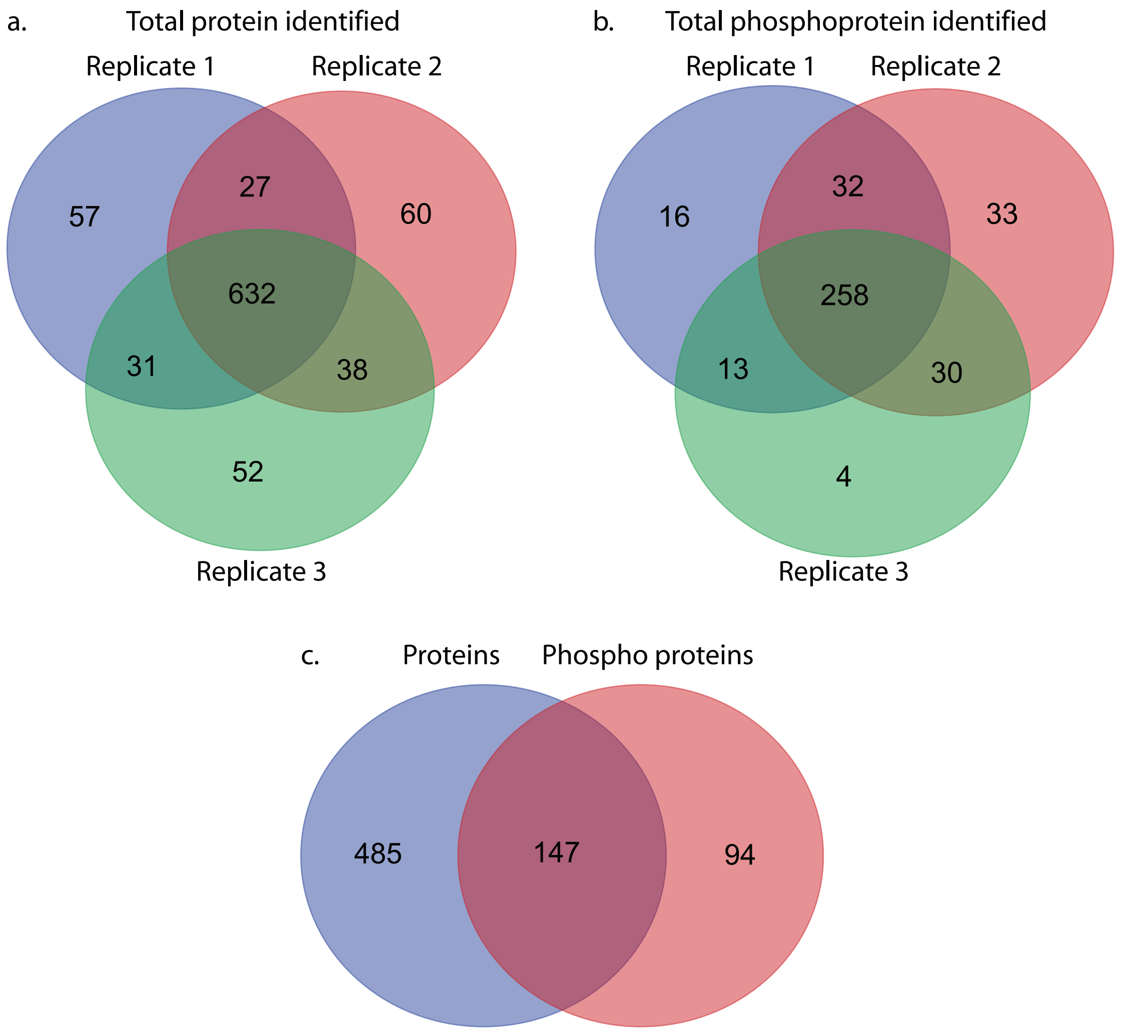


Supplementary Figure6. The details of total proteins and phosphoproteins detected in TMT based q uantitative phosphoproteomics experiment. a. The temporal protein level intensities for 748, 758 and 754 proteins were detected in three technical replicates. The Venn diagram shows the overlaps between replicates. b. The Venn diagram representing the common phosphoproteins detected in three technical replicates c. The overlap between common identified proteins and phosphoproteins (with common phosphopeptides) suggest that the phosphointensity of 147 phosphoproteins could be normalized. 257 phosphopeptides corresponding to these 147 proteins were considered for further analysis.

## Supplementary Figure 7

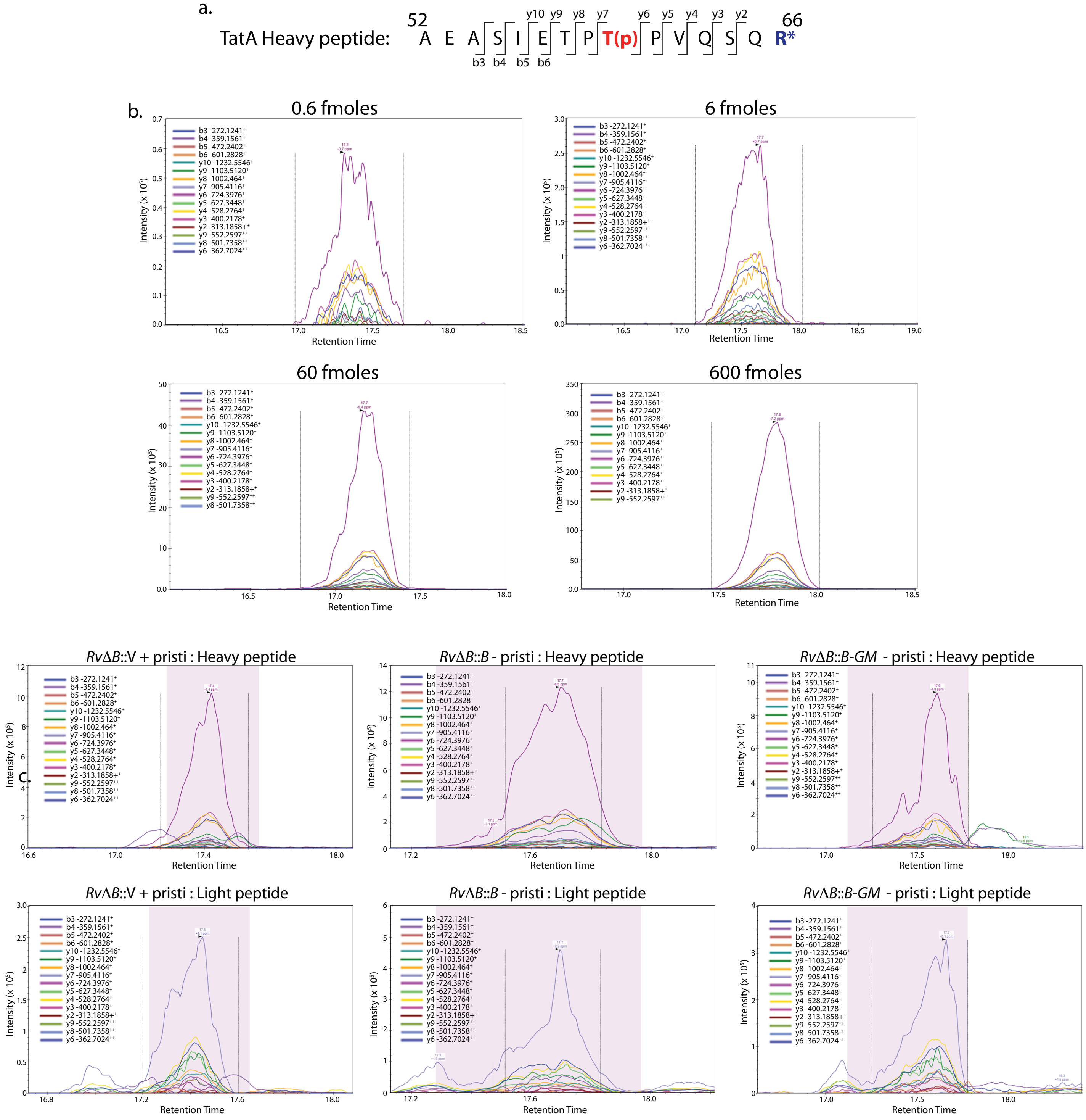


Supplementary Figure7. Standard curve generation for PRM and estimation of TatA T60 phosphorylation a. Synthetic isotopically labelled (SIL) peptide AEASIETPT(p)PVQSQR* with C-terminus 15N and 13C -labeled arginine (Heavy), AEASIETPT(p)PVQSQR* (m/z 852.39, 2+). b and y ion series that was detected in (b) and (c) are shown. b. Graphs displaying extracted chromatograms of SIL peptide at the concentrations depicted above. c. Upper panel: Graphs displaying extracted chromatograms of spiked SIL peptide (Heavy) AEASIETPT(p)PVQSQR* (m/z 852.39, 2+) in the samples. Lower panel: Graphs displaying extracted chromatograms of endogenous peptide (Light) AEASIETPT(p)PVQSQR (m/z 847.39, 2+) in the samples. The retention time and Mass error of the most intense transition are represented above the corresponding peak. The vertical lines on either side of the corresponding peaks represent the integration boundaries.

Supplementary Figure 8: Raw data

anti-PknB blot- Fig 1d

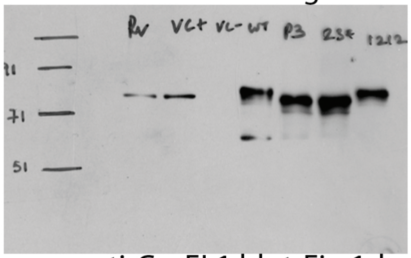

PknB blot: Fig 2c

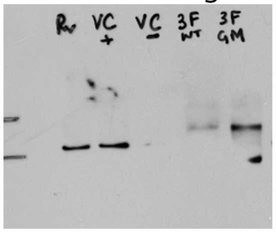

PknB blot: Fig 3b

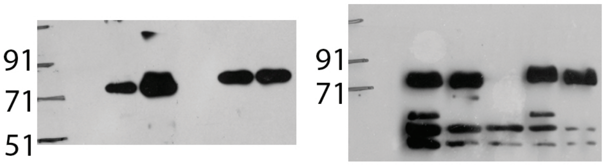


Commassiae Fig 4b

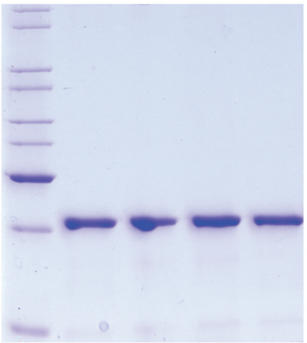

anti-GroEL1 blot-Fig 1d

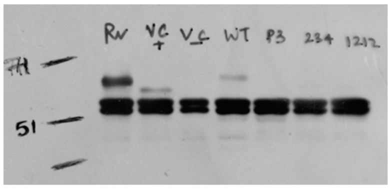

GroEL1 blot: Fig 2c

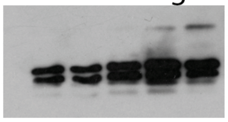

GroEL1 blot: Fig 3b

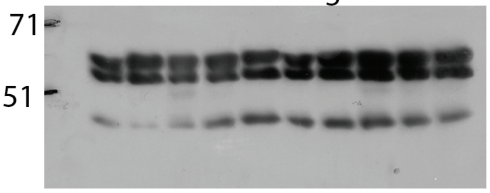


Commassiae Fig 4d

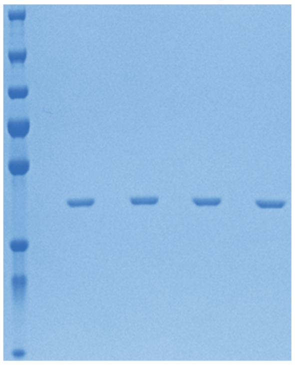

TLC: Fig 4e

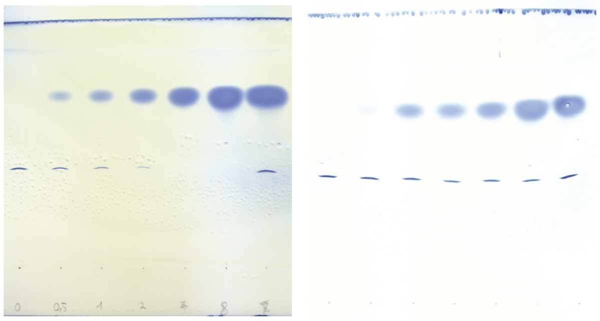

TLC: Fig 4f

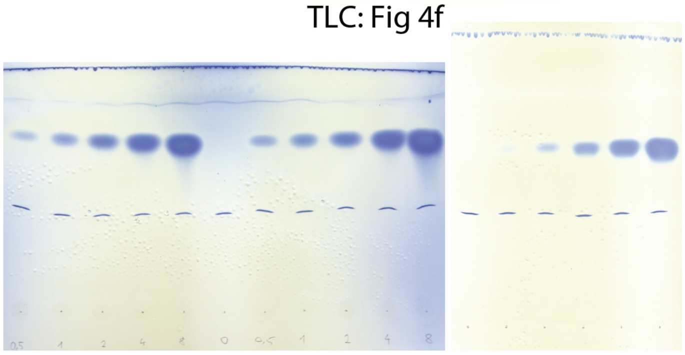

PknB blot: Fig 6b

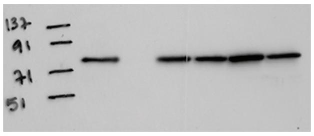

GroEL1 blot: Fig 6b

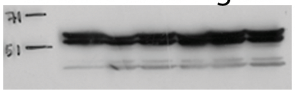

PknB blot: Fig 6F

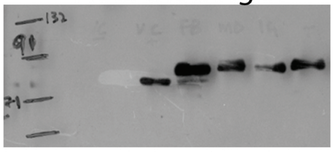

GroEL1 blot: Fig6f

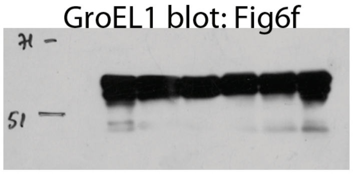

1 2 3 4 5 6

Lanes 4 and 5 were not used for the figure

p-PknB blot: Fig 8b

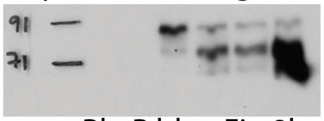

PknB blot: Fig 8b

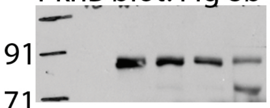


1 2 3 4 5

Lanes 3 and 4 were not used for the figure

p-GarA: Fig 8c Autoradiogram

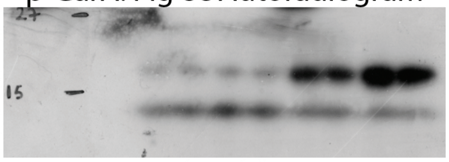

PknB blot: Fig 8c

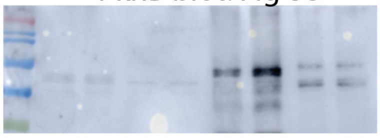

PknB Blot: Fig 8e

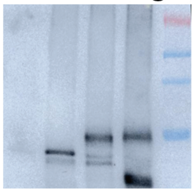

GroEL blot: Fig 8e

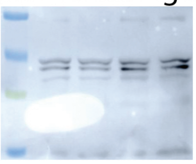

Fig S1c: PknB blot

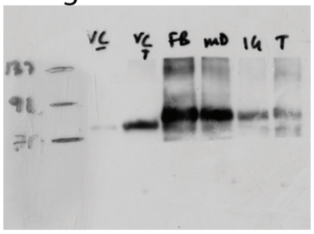

Lanes 1-3 were used

GroEL1: Fig S2c

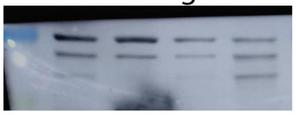

FLAG blot: Fig S2c

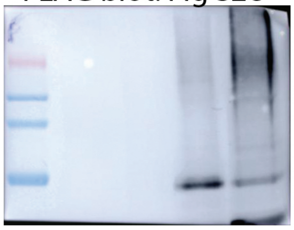

PknB Blot: Fig s2c

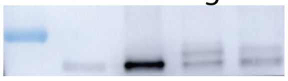

MBP blot: Fig S5a

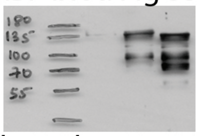

PknB Blot: FigS5a

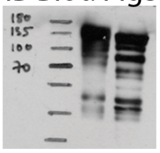

p-PknB blot Fig S5a

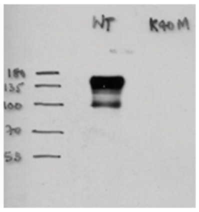

p-PknB blot: Fig S5b lane 1 and 4 were used

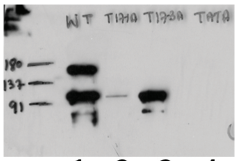

1 2 3 4

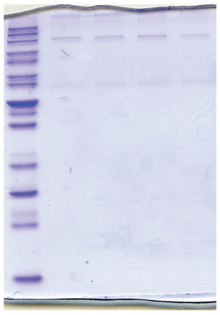

p-PknB blot: Fig S5c

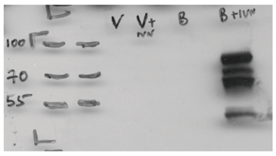

PknB blot: Fig S5c

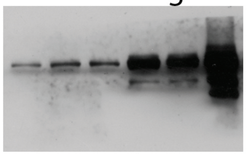

1 2 3 4 5 6

FigS5c: PknB and GroEL1 blot Lanes 2, 3, 4 and 6 were used for the figures.

GroEL1 blot: Fig S5c

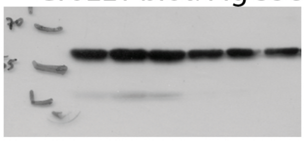

1 2 3 4 5 6
